# Understanding the influence of different proxy perspectives in explaining the difference between self-rated and proxy-rated quality of life in people living with dementia: a systematic literature review and meta-analysis

**DOI:** 10.1007/s11136-024-03660-w

**Published:** 2024-04-24

**Authors:** Lidia Engel, Valeriia Sokolova, Ekaterina Bogatyreva, Anna Leuenberger

**Affiliations:** 1 Monash University Health Economics Group, School of Public Health and Preventive Medicine, Monash University, Level 4, 553 St. Kilda Road, Melbourne, VIC 3004 Australia; 2 School of Health and Social Development, Deakin University, Burwood, VIC Australia

**Keywords:** Quality of Life, Dementia, Outcome measurement, Proxy, Agreement

## Abstract

**Purpose:**

Proxy assessment can be elicited via the proxy-patient perspective (i.e., asking proxies to assess the patient’s quality of life (QoL) as they think the patient would respond) or proxy-proxy perspective (i.e., asking proxies to provide their own perspective on the patient’s QoL). This review aimed to identify the role of the proxy perspective in explaining the differences between self-rated and proxy-rated QoL in people living with dementia.

**Methods:**

A systematic literate review was conducted by sourcing articles from a previously published review, supplemented by an update of the review in four bibliographic databases. Peer-reviewed studies that reported both self-reported and proxy-reported mean QoL estimates using the same standardized QoL instrument, published in English, and focused on the QoL of people with dementia were included. A meta-analysis was conducted to synthesize the mean differences between self- and proxy-report across different proxy perspectives.

**Results:**

The review included 96 articles from which 635 observations were extracted. Most observations extracted used the proxy-proxy perspective (79%) compared with the proxy-patient perspective (10%); with 11% of the studies not stating the perspective. The QOL-AD was the most commonly used measure, followed by the EQ-5D and DEMQOL. The standardized mean difference (SMD) between the self- and proxy-report was lower for the proxy-patient perspective (SMD: 0.250; 95% CI 0.116; 0.384) compared to the proxy-proxy perspective (SMD: 0.532; 95% CI 0.456; 0.609).

**Conclusion:**

Different proxy perspectives affect the ratings of QoL, whereby adopting a proxy-proxy QoL perspective has a higher inter-rater gap in comparison with the proxy-patient perspective.

**Supplementary Information:**

The online version contains supplementary material available at 10.1007/s11136-024-03660-w.

## Background

Quality of life (QoL) has become an important outcome for research and practice but obtaining reliable and valid estimates remains a challenge in people living with dementia [1]. According to the Diagnostic and Statistical Manual of Mental Disorders (DSM-5) criteria [2], dementia, termed as Major Neurocognitive Disorder (MND), involves a significant decline in at least one cognitive domain (executive function, complex attention, language, learning, memory, perceptual-motor, or social cognition), where the decline represents a change from a patient's prior level of cognitive ability, is persistent and progressive over time, is not associated exclusively with an episode of delirium, and reduces a person’s ability to perform everyday activities. Since dementia is one of the most pressing challenges for healthcare systems nowadays [3], it is critical to study its impact on QoL. The World Health Organization defines the concept of QoL as “individuals' perceptions of their position in life in the context of the culture and value systems in which they live and in relation to their goals, expectations, standards, and concerns” [4]. It is a broad ranging concept incorporating in a complex way the persons' physical health, psychological state, level of independence, social relationships, personal beliefs, and their relationships to salient features of the environment.

Although there is evidence that people with mild to moderate dementia can reliably rate their own QoL [5], as the disease progresses, there is typically a decline in memory, attention, judgment, insight, and communication that may compromise self-reporting of QoL [6]. Additionally, behavioral symptoms, such as agitation, and affective symptoms, such as depression, may present another challenge in obtaining self-reported QoL ratings due to emotional shifts and unwillingness to complete the assessment [7]. Although QoL is subjective and should ideally be assessed from an individual’s own perspective [8], the decline in cognitive function emphasizes the need for proxy-reporting by family members, health professionals, or care staff who are asked to report on behalf of the person with dementia. However, proxy-reports are not substitutable for self-reports from people with dementia, as they offer supplementary insights, reflecting the perceptions and viewpoints of people surrounding the person with dementia [9].

Previous research has consistently highlighted a disagreement between self-rated and proxy-rated QoL in people living with dementia, with proxies generally providing lower ratings (indicating poorer QoL) compared with person’s own ratings [8, 10–12]. Impairment in cognition associated with greater dementia severity has been found to be associated with larger difference between self-rating and proxy-rating obtained from family caregivers, as it becomes increasingly difficult for severely cognitively impaired individuals to respond to questions that require contemplation, introspection, and sustained attention [13, 14]. Moreover, non-cognitive factors, such as awareness of disease and depressive symptoms play an important role when comparing QoL ratings between individuals with dementia and their proxies [15]. Qualitative evidence has also shown that people with dementia tend to compare themselves with their peers, whereas carers make comparisons with how the person used to be in the past [9]. The disagreement between self-reported QoL and carer proxy-rated QoL could be modulated by some personal, cognitive or relational factors, for example, the type of relationship or the frequency of contact maintained, person’s cognitive status, carer’s own feeling about dementia, carer’s mood, and perceived burden of caregiving [14, 16]. Disagreement may also arise from the person with dementia’s problems to communicate symptoms, and proxies’ inability to recognize certain symptoms, like pain [17], or be impacted by the amount of time spent with the person with dementia [18]. This may also prevent proxies to rate accurately certain domains of QoL, with previous evidence showing higher level of agreement for observable domains, such as mobility, compared with less observable domains like emotional wellbeing [8]. Finally, agreement also depends on the type of proxy (i.e., informal/family carers or professional staff) and the nature of their relationship, for instance, proxy QoL scores provided by formal carers tend to be higher (reflecting better QoL) compared to the scores supplied by family members [19, 20]. Staff members might associate residents’ QoL with the quality of care delivered or the stage of their cognitive impairment, whereas relatives often focus on comparison with the person’s QoL when they were younger, lived in their own home and did not have dementia [20].

What has been not been fully examined to date is the role of different proxy perspectives employed in QoL questionnaires in explaining disagreement between self-rated and proxy-rated scores in people with dementia. Pickard et al. (2005) have proposed a conceptual framework for proxy assessments that distinguish between the proxy-patient perspective (i.e., asking proxies to assess the patient’s QoL as they think the patient would respond) or proxy-proxy perspective (i.e., asking proxies to provide their own perspective on the patient’s QoL) [21]. In this context, the *intra-proxy gap* describes the differences between proxy-patient and proxy-proxy perspective, whereas the *inter-rater gap* is the difference between self-report and proxy-report [21].

Existing generic and dementia-specific QoL instruments specify the perspective explicitly in their instructions or imply the perspective indirectly in their wording. For example, the instructions of the Dementia Quality of Life Measure (DEMQOL) asks proxies to *give the answer they think their relative would give* (i.e., proxy-patient perspective) [22], whereas the family version of the Quality of Life in Alzheimer’s Disease (QOL-AD) instructs the proxies to *rate their relative’s current situation as they (the proxy) see it* (i.e., proxy-proxy perspective) [7]. Some instruments, like the EQ-5D measures, have two proxy versions for each respective perspective [23, 24]. The Adult Social Care Outcome Toolkit (ASCOT) proxy version, on the other hand, asks proxies to complete the questions from both perspectives, from their *own opinion* and *how they think the person would answer* [25].

QoL scores generated using different perspectives are expected to differ, with qualitative evidence showing that carers rate the person with dementia’s QoL lower (worse) when instructed to comment from their own perspective than from the perspective of the person with dementia [26]. However, to our knowledge, no previous review has fully synthesized existing evidence in this area. Therefore, we aimed to undertake a systematic literature review to examine the role of different proxy-assessment perspectives in explaining differences between self-rated and proxy-rated QoL in people living with dementia. The review was conducted under the hypothesis that the difference in QoL estimates will be larger when adopting the proxy-proxy perspective compared with proxy-patient perspective.

## Methods

The review was registered with the International Prospective Register of Systematic Reviews (CRD42022333542) and followed the Preferred Reporting Items System for Systematic Reviews and Meta-Analysis (PRISMA) guidelines (see Appendix 1) [27].

### Search strategy

This review used two approaches to obtain literature. First, primary articles from an existing review by Roydhouse et al. were retrieved [28]. The review included studies published from inception to February 2018 that compared self- and proxy-reports. Studies that focused explicitly on Alzheimer’s Disease or dementia were retrieved for the current review. Two reviewers conducted a full-text review to assess whether the eligibility criteria listed below for the respective study were met. An update of the Roydhouse et al. review was undertaken to capture more recent studies. The search strategy by Roydhouse et al. was amended and covered studies published after January 1, 2018, and was limited to studies within the context of dementia. The original search was undertaken over a three-week period (17/11/2021–9/12/2021) and then updated on July 3, 2023. Peer-reviewed literature was sourced from MEDLINE, CINAHL, and PsycINFO databases via EBSCOHost as well as EMBASE. Four main search term categories were used: (1) proxy terms (i.e., care*-report*), (2) QoL/ outcome terms (i.e., ‘quality of life’), (3) disease terms (i.e., ‘dementia’), and (4) pediatric terms (i.e., ‘pediatric*’) (for exclusion). Keywords were limited to appear in titles and abstracts only, and MeSH terms were included for all databases. A list of search strategy can be found in Appendix 2. The first three search term categories were searched with AND, and the NOT function was used to exclude pediatric terms. A limiter was applied in all database searches to only include studies with human participants and articles published in English.

### Selection criteria

Studies from all geographical locations were included in the review if they (1) were published in English in a peer-reviewed journal (conference abstracts, dissertations, a gray literature were excluded); (2) were primary studies (reviews were excluded); (3) clearly defined the disease of participants, which were limited to Alzheimer’s disease or dementia; (4) reported separate QoL scores for people with dementia (studies that included mixed populations had to report a separate QoL score for people with dementia to be considered); (5) were using a standardized and existing QoL instrument for assessment; and (6) provided a mean self-reported and proxy-reported QoL score for the same dyads sample (studies that reported means for non-matched samples were excluded) using the same QoL instrument.

### Screening

Four reviewers (LE, VS, KB, AL) were grouped into two groups who independently screened the 179 full texts from the Roydhouse et. al (2022) study that included Alzheimer’s disease or dementia patients. If a discrepancy within the inclusion selection occurred, articles were discussed among all the reviewers until a consensus was reached. Studies identified from the database search were imported into EndNote [29]. Duplicates were removed through EndNote and then uploaded to Rayyan [30]. Each abstract was reviewed by two independent reviewers (any two from four reviewers). Disagreements regarding study inclusions were discussed between all reviewers until a consensus was reached. Full-text screening of each eligible article was completed by two independent reviewers (any two from four reviewers). Again, a discussion between all reviewers was used in case of disagreements.

### Data extraction

A data extraction template was created in Microsoft Excel. The following information were extracted if available: country, study design, study sample, study setting, dementia type, disease severity, Mini-Mental Health State Exam (MMSE) score details, proxy type, perspective, living arrangements, QoL assessment measure/instrument, self-reported scores (mean, SD), proxy-reported scores (mean, SD), and agreement statistics. If a study reported the mean (SD) for the total score as well as for specific QoL domains of the measure, we extracted both. If studies reported multiple scores across different time points or subgroups, we extracted all scores. For interventional studies, scores from both the intervention group and the control group were recorded. In determining the proxy perspective, we relied on authors’ description in the article. If the perspective was not explicitly stated, we adopted the perspective of the instrument developers; where more perspectives were possible (e.g., in the case of the EQ-5D measures) and the perspective was not explicitly stated, it was categorized as ‘undefined.’ For agreement, we extracted the Intraclass Correlation Coefficient (ICC), a reliability index that reflects both degree of correlation and agreement between measurements of continuous variables. While there are different forms of ICC based on the model (1-way random effects, 2-wy random effects, or 2-way fixed effects), the type (single rater/measurement or the mean *k* raters/measurements), and definition of relationship [31], this level of information was not extracted due to insufficient information provided in the original studies. Values for ICC range between 0 and 1, with values interpreted as poor (less than 0.5), moderate (0.5–0.75), good (0.75–0.9), and excellent (greater than 0.9) reliability between raters [31].

### Data synthesis and analysis

Characteristics of studies were summarized descriptively. Self-reported and proxy-reported means and SD were extracted from the full texts and the mean difference was calculated (or extracted if available) for each pair. Studies that reported median values instead of mean values were converted using the approach outlined by Wan et al. (2014) [32]. Missing SDs (5 studies, 20 observations) were obtained from standard errors or confidence intervals reported following the Cochrane guidelines [33]. Missing SDs (6 studies, 29 observations) in studies that only presented the mean value without any additional summary statistics were imputed using the prognostic method [34]. Thereby, we predicted the missing SDs by calculating the average SDs of observed studies with full information by the respective measure and source (self-report versus proxy-report).

A meta-analysis was performed in Stata (17.1 Stata Corp LLC, College Station, TX) to synthesize mean differences between self- and proxy-reported scores across different proxy perspectives. First, the pooled raw mean differences were calculated for each QoL measure separately, given differences in scales between measures. Secondly, we calculated the pooled standardized mean difference (SMD) for all studies stratified by proxy type (family carer, formal carers, mixed), dementia severity (mild, moderate, severe), and living arrangement (residential/institutional care, mixed). SMD accounts for the use of different measurement scales, where effect sizes were estimated using Cohen’s d. Random-effects models were used to allow for unexplained between-study variability based on the restricted maximum-likelihood (REML) estimator. The percentage of variability attributed to heterogeneity between the studies was assessed using the *I*^*2*^ statistic; an *I*^*2*^ of 0%-40% represents possibly unimportant heterogeneity, 30–60% moderate heterogeneity, 50–90% substantial heterogeneity, and 75%-100% considerable heterogeneity [35]. Chi-squared statistics (χ^2^) provided evidence of heterogeneity, where a *p*-value of 0.1 was used as significance level. For studies that reported agreement statistics, based on ICC, we also ran a forest plot stratified by the study perspective. We also calculated Q statistic (Cochran’s test of homogeneity), which assesses whether observed differences in results are compatible with chance alone.

### Risk of bias and quality assessment

The quality of studies was assessed using the using a checklist for assessing the quality of quantitative studies developed by Kmet et al. (2004) [36]. The checklist consists of 14 items and items are scored as ‘2’ (yes, item sufficiently addressed), ‘1’ (item partially addressed), ‘0’ (no, not addressed), or ‘not applicable.’ A summary score was calculated for each study by summing the total score obtained across relevant items and dividing by the total possible score. Scores were adjusted by excluding items that were not applicable from the total score. Quality assessment was undertaken by one reviewer, with 25% of the papers assessed independently by a second reviewer.

## Results

**Fig. 1:**
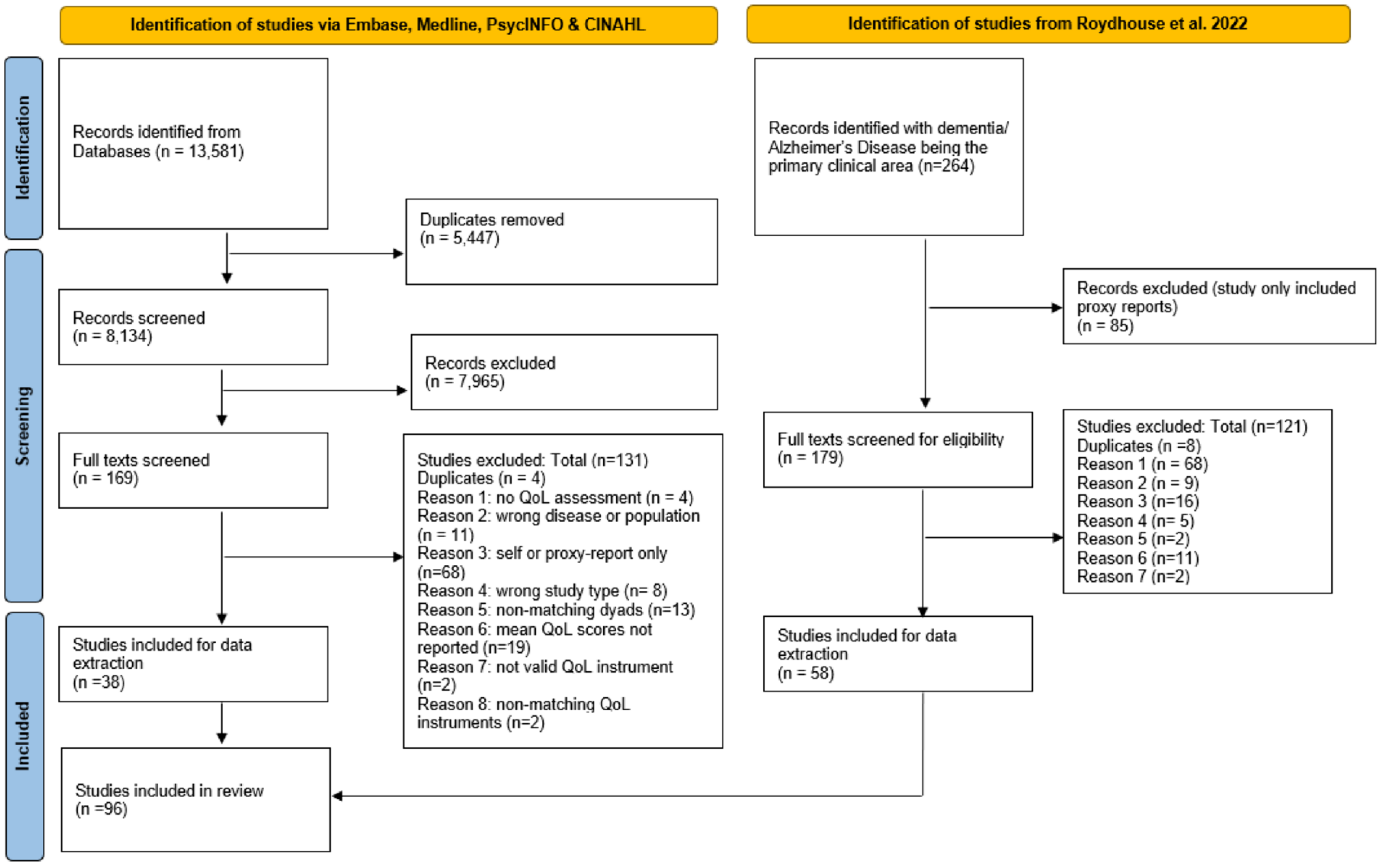
PRISMA 2020 flow diagram

The PRISMA diagram in Fig. 1 shows that after the abstract and full-text screening, 38 studies from the database search and 58 studies from the Roydhouse et al. (2022) review were included in this review—a total of 96 studies. A list of all studies included and their characteristics can be found in Appendix 3.

### General study characteristics

The 96 articles included in the review were published between 1999 and 2023 from across the globe; most studies (36%) were conducted in Europe. People with dementia in these studies were living in the community (67%), residential/institutional care (15%), as well as mixed dwelling settings (18%). Most proxy-reports were provided by family carers (85%) and only 8 studies (8%) included formal carers. The mean MMSE score for dementia and Alzheimer’s participants was 18.77 (SD = 4.34; *N* = 85 studies), which corresponds to moderate cognitive impairment [37]. Further characteristics of studies included are provided in Table 1. The quality of studies included (see Appendix 4) was generally very good, scoring on average 91% (SD: 9.1) with scores ranging from 50 to 100%.

**Table 1:** Characteristics of studies included (*n* = 96)

	N (%)
*Country/Continent*	
Europe UK	35 (36.46) 14 (14.6)
US	12 (12.5)
Brazil	13 (13.5)
Australia	7 (7.3)
Japan	5 (5.2)
Canada	2 (2.1)
Turkey	3 (3.1)
Taiwan	2 (2.1)
China	1 (1.0)
Singapore	1 (1.0)
Multiple Global Study Sites	1 (1.0)
*Setting*	
Community-dwelling	64 (66.67)
Residential/institutional care	14 (14.58)
Mixed	18 (17.75)
*Proxy type*	
Formal	8 (8.3)
Family Carers	82 (85.42)
Mixed	5 (5.21)
Undefined	1 (1.04)
*MMSE Score*	
MMSE mean score (SD), min: 9; max: 28.9 *N* = 85	18.77 (4.34)

*MMSE* Mini-Mental State Examination. MMSE scores range from 0 to 30, where 27 to 30 = no cognitive impairment; 21 to 26 = mild cognitive impairment; and 10 to 20 = moderate cognitive impairment; less than 10 = severe cognitive impairment

### Quality of life measure and proxy perspective used

A total of 635 observations were recorded from the 96 studies. The majority of studies and observations extracted assumed the proxy-proxy perspective (77 studies, 501 observations), followed by the proxy-patient perspective (18 studies, 62 observations), with 18 studies (72 observations) not clearly defining the perspective. Table 2 provides a detailed overview of number of studies and observations across the respective QoL measures and proxy perspectives. Two studies (14 observations) adopted both perspectives within the same study design: one using the QOL-AD measure [5] and the second study exploring the EQ-5D-3L and EQ VAS [38]. Overall, the QOL-AD was the most often used QoL measure, followed by the EQ-5D and DEMQOL. Mean scores for specific QoL domains were accessible for the DEMQOL and QOL-AD. However, only the QOL-AD provided domain-specific mean scores from both proxy perspectives.

**Table 2:** Overview of number of studies (number of observations) by measure and proxy perspective

	Proxy-proxy	Proxy-patient	Undefined
DEMQOL		10 (18)	
DEMQOL domains		2 (7)	
DEMQOL-U		4 (11)	
DQI			1 (1)
DQoL	1 (5)		1 (18)
EQ-5D			1 (2)
EQ-5D-3L	3 (13)	2 (6)	8 (17)
EQ-5D-5L			5 (12)
EQ VAS	3 (5)	1 (3)	7 (14)
HUI3			1 (1)
ICECAP-O			2 (4)
QOL-AD 13 Item	67 (130)	3 (4)	
QOL-AD 13 Item domains	17 (299)	1 (13)	
QOL-AD 10 Item	1 (3)		
QOL-AD 12 Item	1 (1)		
QOL-AD 15 Item	5 (15)		
QOL-AD 15 Item domains	1 (30)		
QWB			1 (1)
SF-12 MSC SF-12 PCS			1 (1) 1 (1)
TOTAL	77 (501)	18 (62)	18 (72)

The total number of studies does not add up to 96 because some studies used multiple QoL measures that adopted different perspectives

### Mean scores and mean differences by proxy perspective and QoL measure

The raw mean scores for self-reported and proxy-reported QoL scores are provided in the Supplementary file 2. The pooled raw mean difference by proxy perspective and measure is shown in Table 3. Regardless of the perspective adopted and the QoL instrument used, self-reported scores were higher (indicating better QoL) compared with proxy-reported scores, except for the DEMQOL, where proxies reported better QoL than people with dementia themselves. Most instruments were explored from one perspective, except for the EQ-5D-3L, EQ VAS, and QOL-AD, for which mean differences were available for both perspectives. For these three measures, mean differences were smaller when adopting the proxy-patient perspective compared with proxy-proxy perspective, although mean scores for the QOL-AD were slightly lower from the proxy-proxy perspective. *I*^*2*^ statistics indicate considerable heterogeneity (I^2^ > 75%) between studies. Mean differences by specific QoL domains are provided in Appendix 5, but only for the QOL-AD measure that was explored from both perspectives. Generally, mean differences appeared to be smaller for the proxy-proxy perspective than the proxy-patient perspective across all domains, except for ‘physical health’ and ‘doing chores around the house.’ However, results need to be interpreted carefully as proxy-patient perspective scores were derived from only one study.

**Table 3:** Pooled raw mean difference by proxy perspective and measure

	Proxy-proxy					Proxy-patient					Undefined				
	N(O)	Self	Proxy	MD (95% CI)	*I*^*2*^	N(O)	Self	Proxy	MD (95% CI)	*I*^*2*^	N(O)	Self	Proxy	MD (95% CI)	*I*^*2*^
DEMQOL	−	−	−	−	−	10 (18)	92.24	94.30	−2.03 (−4.38; −0.32)	85.92	−	−	−	−	−
DEMQOL-U	−	−	−	−	−	4 (11)	0.74	0.69	0.04 (−0.02; 0.097)	96.09	−	−	−	−	−
DQI	−	−	−	−	−	−	−	−	−	−	1 (1)	0.81	0.97	−0.16 (−0.21; −0.11)	−
DQoL	1 (5)	3.67	3.15	0.512 (0.08; 0.39)	51.49	−	−	−	−	−	1 (18)	3.57	3.41	0.136 (−0.04; 0.31)	72.78
EQ-5D	−	−	−	−	−	−	−	−	−	−	1 (2)	0.669	0.429	0.24 (0.07; 0.41)	90.78
EQ-5D-3L	3 (13)	0.723	0.554	0.17 (0.13; 0.20)	95.39	2 (6)	0.626	0.546	0.08 (0.03; 0.12)	95.75	8 (17)	0.776	0.591	0.186 (0.13; 0.24)	97.10
EQ−5D−5L	−	−	−	−	−	−	−	−	−	−	5 (12)	0.808	0.605	0.19 (0.13; 0.25)	96.02
EQ−VAS	3 (5)	69.10	64.43	4.53 (−0.33; 9.39)	98.43	1 (3)	65.47	65.24	0.20 (−0.42; 0.82)	0	7 (14)	71.68	57.74	12.24 (9.77; 14.71)	73.55
HUI	−	−	−	−	−	−	−	−	−	−	1 (1)	0.73	0.23	0.50 (0.36; 0.64)	−
ICECAP-O	−	−	−	−	−	−	−	−	−	−	2 (4)	0.666	0.559	0.11 (0.09; 0.12)	46.16
QOL-AD 13 Item	67 (130)	34.70	30.37	4.18 (3.62; 4.74)	99.10	3 (4)	35.67	31.37	4.30 (3.13; 5.47)	41.46	−	−	−	−	−
QOL-AD 10 Item	1 (3)	36.13	29.57	6.38 (4.25; 8.51)	52.81	−	−	−	−	−	−	−	−	−	−
QOL-AD 12 Item	1 (1)	34.71	28.41	6.30 (5.05; 7.51)	−	−	−	−	−	−	−	−	−	−	−
QOL-AD 15 Item	5 (15)	2.97	2.49	0.48 (0.39; 0.57)	66.01	−	−	−	−	−	−	−	−	−	−
QWB	−	−	−	−	−	−	−	−	−	−	1 (1)	0.6	0.42	0.180 (0.06; 0.30)	−
SF-12 PCS	−	−	−	−	−	−	−	−	−	−	1 (1)	45.74	42.02	3.720 (−3.39; 10.83)	−
SF-12 MSC	−	−	−	−	−	−	−	−	−	−	1 (1)	49.38	49.27	0.11 (−6.43; 6.65)	−

*N(O)* number of studies (number of observations); *MD* = mean difference between self- and proxy-reported scores.* I*^*2*^ statistic

### Standardized mean differences by proxy perspective, stratified by proxy type, dementia severity, and living arrangement

Table 4 provides the SMD by proxy perspective, which adjusts for the different QoL measurement scales. Findings suggest that adopting the proxy-patient perspective results in lower SMDs (SMD: 0.250; 95% CI 0.116; 0.384) compared with the proxy-proxy perspective (SMD: 0.532; 95% CI 0.456; 0.609). The largest SMD was recorded for studies that did not define the study perspective (SMD: 0.594; 95% CI 0.469; 0.718). A comparison by different proxy types (formal carers, family carers, and mixed proxies) revealed some mixed results. When adopting the proxy-proxy perspective, the largest SMD was found for family carers (SMD: 0.556; 95% CI 0.465; 0.646) compared with formal carers (SMD: 0.446; 95% CI 0.305; 0.586) or mixed proxies (SMD: 0.335; 95% CI 0.211; 0.459). However, the opposite relationship was found when the proxy-patient perspective was used, where the smallest SMD was found for family carers compared with formal carers and mixed proxies. The SMD increased with greater level of dementia severity, suggesting a greater disagreement. However, compared with the proxy-proxy perspective, where self-reported scores were greater (i.e., better QoL) than proxy-reported scores across all dementia severity levels, the opposite was found when adopting the proxy-patient perspective, where proxies reported better QoL than people with dementia themselves, except for the severe subgroup. No clear trend was observed for different living settings, although the SMD appeared to be smaller for people with dementia living in residential care compared with those living in the community.

**Table 4:** Pooled standardized mean difference by proxy perspective, stratified by proxy type, dementia severity, and living arrangement

	Proxy-proxy			Proxy-patient			Undefined			Group difference χ^2^
	N*	SMD (95% CI)	*I*^*2*^ (%)	N*	SMD (95% CI)	*I*^*2*^ (%)	N*	SMD (95% CI)	*I*^*2*^ (%)	
*TOTAL (n* = *635)*	501	0.532 (0.456; 0.609)	98.49	62	0.250 (0.116; 0.384)	95.02	72	0.594 (0.469; 0.718)	95.08	16.09 (*p* ≤ 0.001)
*Proxy type*										
Family carer (*n* = 535)	418	0.556 (0.465; 0.646)	98.40	46	0.228 (0.093; 0.363)	91.53	71	0.621 (0.507; 0.734)	93.90	21.43 (*p* ≤ 0.001)
Formal carer (*n* = 72)	64	0.446 (0.305; 0.586)	96.20	8	0.232 (−0.222; 0.685)	98.37	−	−	−	0.78 (*p* = 0.377)
Mixed (*n* = 25)	17	0.335 (0.211; 0.459)	97.06	8	0.378 (−0.173; 0.929)	97.84	−	−	−	0.02 (*p* = 0.881)
*Dementia severity*										
Mild (*n* = 46)	26	0.389 (0.223; 0.555)	85.94	3	−0.085 (−0.603; 0.433)	91.32	17	0.323 (0.118; 0.527)	84.02	2.94 (*p* = 0.230)
Moderate (*n* = 31)	24	0.736 (0.580; 0.891)	82.19	2	−0.128 (−0.376; 0.119)	0	5	0.824 (0.435; 1.214)	95.43	36.18 (*p* ≤ 0.001)
Severe (*n* = 7)	4	1.277 (0.979; 1.575)	88.21	1	0.534 (0.273; 0.795)	−	2	1.335 (0.637; 2.033)	98.49	15.17 (*p* = 0.001)
*Setting*										
Residential care (*n* = 99)	77	0.550 (0.433; 0.667)	89.52	15	0.282 (−0.086; 0.650)	98.34	7	0.519 (0.392; 0.645)	79.10	1.85 (*p* = 0.396)
Community (*n* = 402)	324	0.551 (0.433; 0.669)	98.55	37	0.378 (0.243; 0.513)	89.54	41	0.458 (0.280; 0.637)	94.01	3.61 (*p* = 0.164)
Mixed (*n* = 134)	100	0.483 (0.401; 0.566)	97.35	10	−0.303 (−0.388; −0.218)	0	24	0.818 (0.619; 1.017)	94.64	214.64 (*p* ≤ 0.001)

N* = number of observations

### Direct proxy perspectives comparison studies

Two studies assessed both proxy perspectives within the same study design. Bosboom et al. (2012) found that compared with self-reported scores (mean: 34.7; SD: 5.3) using the QOL-AD, proxy scores using the proxy-patient perspective were closer to the self-reported scores (mean: 32.1; SD: 6.1) compared with the proxy-proxy perspective (mean: 29.5; SD: 5.4) [5]. Similar findings were reported by Leontjevas et al. (2016) using the EQ-5D-3L, including the EQ VAS, showing that the inter-proxy gap between self-report (EQ-5D-3L: 0.609; EQ VAS: 65.37) and proxy-report was smaller when adopting the proxy-patient perspective (EQ-5D-3L: 0.555; EQ VAS: 65.15) compared with the proxy-proxy perspective (EQ-5D-3L: 0.492; EQ VAS: 64.42) [38].

### Inter-rater agreement (ICC) statistics

**Fig. 2:**
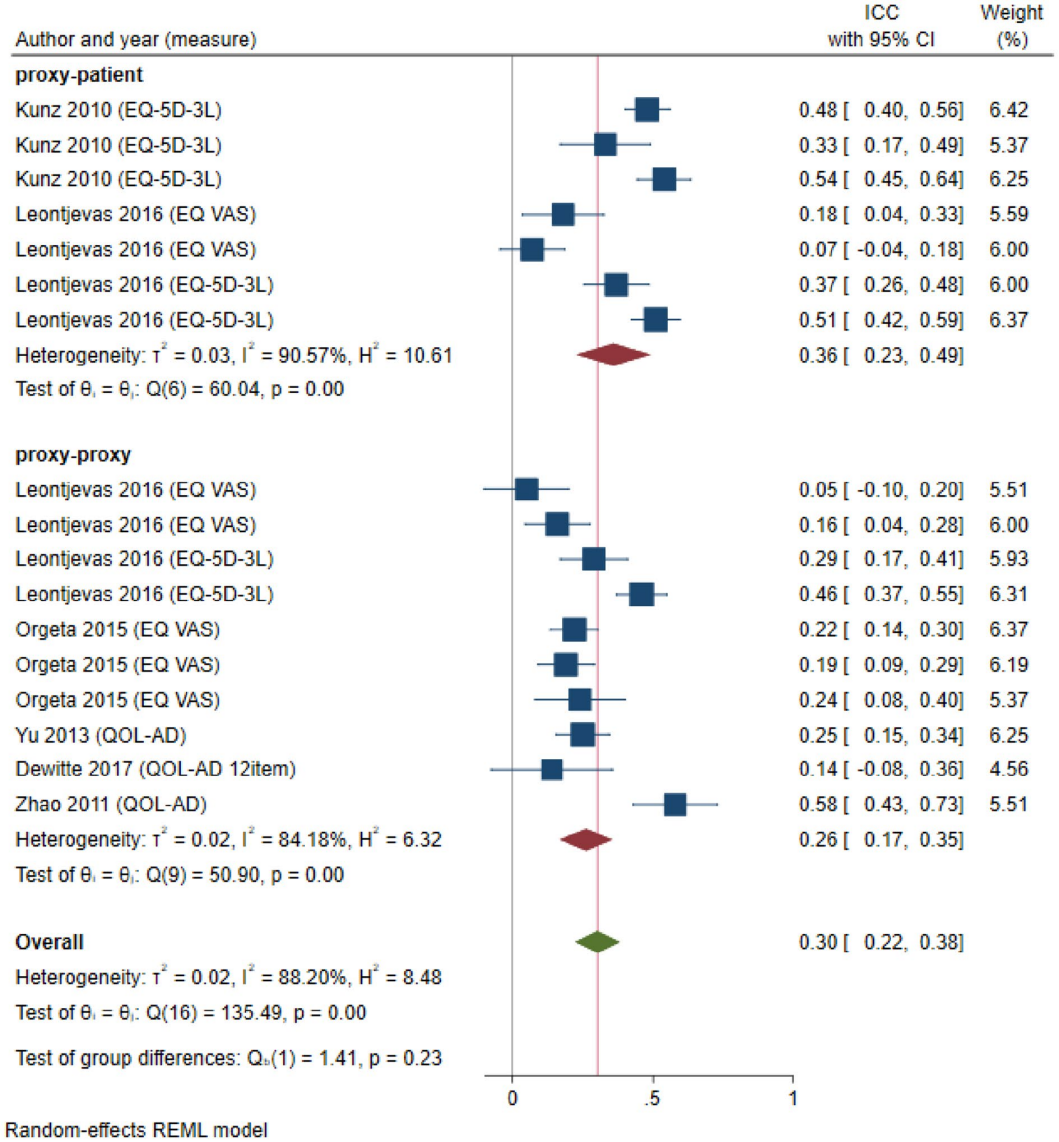
Forest plot depicting study-specific and overall ICC estimates by study perspective

Six studies reported agreement statistics based on ICC, from which we extracted 17 observations that were included in the meta-analysis. Figure 2 shows the study-specific and overall estimates of ICC by the respective study perspective. The heterogeneity between studies was high (*I*^2^ = 88.20%), with a *Q* test score of 135.49 (*p* < 0.001). While the overall ICC for the 17 observations was 0.3 (95% CI 0.22; 0.38), indicating low agreement, the level of agreement was slightly better when adopting a proxy-patient perspective (ICC: 0.36, 95% CI 0.23; 0.49) than a proxy-proxy perspective (ICC: 0.26, 95% CI 0.17; 0.35).

## Discussion

While previous studies highlighted a disagreement between self-rated and proxy-rated QoL in people living with dementia, this review, for the first time, assessed the role of different proxy perspectives in explaining the inter-rater gap. Our findings align with the baseline hypothesis and indicate that QoL scores reported from the proxy-patient perspective are closer to self-reported QoL scores than the proxy-proxy perspective, suggesting that the proxy perspective does impact the inter-rater gap and should not be ignored. This finding was observed across different analyses conducted in this review (i.e., pooled raw mean difference, SMD, ICC analysis), which also confirms the results of two previous primary studies that adopted both proxy perspectives within the same study design [5, 38]. Our findings emphasize the need for transparency in reporting the proxy perspective used in future studies, as it can impact results and interpretation. This was also noted by the recent ISPOR Proxy Task Force that developed a checklist of considerations when using proxy-reporting [39]. While consistency in proxy-reports is desirable, it is crucial to acknowledge that each proxy perspective holds significance in future research, depending on study objectives. It is evident that both proxy perspectives offer distinct insights—one encapsulating the perspectives of people with dementia, and the other reflecting the viewpoints of proxies. Therefore, in situations where self-report is unattainable due to advanced disease severity and the person’s perspective on their own QoL assessment is sought, it is recommended to use the proxy-patient perspective. Conversely, if the objective of future research is to encompass the viewpoints of proxies, opting for the proxy-proxy perspective is advisable. However, it is important to note that proxies may deviate from instructed perspectives, requiring future qualitative research to examine the adherence to proxy perspectives. Additionally, others have argued that proxy-reports should not substitute self-reports, and only serve as supplementary sources alongside patient self-reports whenever possible [9].

This review considered various QoL instruments, but most instruments adopted one specific proxy perspective, limiting detailed analyses. QoL instruments differ in their scope (generic versus disease-specific) as well as coverage of QoL domains. The QOL-AD, an Alzheimer's Disease-specific measure, was commonly used. Surprisingly, for this measure, the mean differences between self-reported and proxy-reported scores were smaller using the proxy-proxy perspective, contrary to the patterns observed with all other instruments. This may be due to the lack of studies reporting QOL-AD proxy scores from the proxy-patient perspective, as the study by Bosboom et al. (2012) found the opposite [5]. Previous research has also suggested that the inter-rater gap is dependent on the QoL domains and that the risk of bias is greater for more ‘subjective’ (less observable) domains such as emotions, feelings, and moods in comparison with observable, objective areas such as physical domains [8, 40]. However, this review lacks sufficient observations for definitive results on QoL dimensions and their impact on self-proxy differences, emphasizing the need for future research in this area.

With regard to proxy type, there is an observable trend suggesting a wider inter-rater gap when family proxies are employed using the proxy-proxy perspective, in contrast to formal proxies. This variance might be attributed to the use of distinct anchoring points; family proxies tend to assess the individual's QoL in relation to their past self before having dementia, while formal caregivers may draw comparisons with other individuals with dementia under their care [41]. However, the opposite was found when the proxy-patient perspective was used, where family proxies scores seemed to align more closely with self-reported scores, resulting in lower SMD scores. This suggests that family proxies might possess a better ability to empathize with the perspective of the person with dementia compared to formal proxies. Nonetheless, it is important to interpret these findings cautiously, given the relatively small number of observations for formal caregiver reports. Additionally, other factors such as emotional connection, caregiver burden, and caregiver QoL may also impact proxy-reports by family proxies [14, 16] that have not been explored in this review.

Our review found that the SMD between proxy and self-report increased with greater level of dementia severity, contrasting a previous study, which showed that cognitive impairment was not the primary factor that accounted for the differences in the QoL assessments between family proxies and the person with dementia [15]. However, it is noteworthy that different interpretations and classifications were used across studies to define mild, moderate, and severe dementia, which needs to be considered. Most studies used MMSE to define dementia severity levels. Given the MMSE’s role as a standard measure of cognitive function, the study findings are considered generalizable and clinically relevant for people with dementia across different dementia severity levels. When examining the role of the proxy perspective by level of severity, we found that compared with the proxy-proxy perspective, where self-reported scores were greater than proxy-reported scores across all dementia severity levels, the proxy-patient perspective yielded the opposite results, and proxies reported better QoL than people with dementia themselves, except for the severe subgroup. It is possible that in the early stages of dementia, the person with dementia has a greater awareness of increasing deficits, coupled with denial and lack of acceptance, leading to a more critical view of their own QoL than how proxies think they would rate their QoL. However, future studies are warranted, given the small number of observations adopting the proxy-patient perspective in our review.

The heterogeneity observed in the studies included was high, supporting the use of random-effects meta-analysis. This is not surprising given the diverse nature of studies included (i.e., RCTs, cross-sectional studies), differences in the population (i.e., people living in residential care versus community-dwelling people), mixed levels of dementia severity, and differences between instruments. While similar heterogeneity was observed in another review on a similar topic [42], our presentation of findings stratified by proxy type, dementia severity, and living arrangement attempted to account for such differences across studies.

### Limitations and recommendations for future studies

Our review has some limitations. Firstly, proxy perspectives were categorized based on the authors' descriptions, but many papers did not explicitly state the perspective, which led to the use of assumptions based on instrument developers. Some studies may have modified the perspective's wording without reporting it. Due to lack of resources, we did not contact the authors of the original studies directly to seek clarification around the proxy perspective adopted. Regarding studies using the EQ-5D, which has two proxy perspectives, some studies did not specify which proxy version was used, suggesting the potential use of self-reported versions for proxies. In such cases, the proxy perspective was categorized as undefined. Despite accounting for factors like QoL measure, proxy type, setting, and dementia severity, we could not assess the impact of proxy characteristics (e.g., carer burden) or dementia type due to limited information provided in the studies. We also faced limitations in exploring the proxy perspective by QoL domains due to limited information. Further, not all studies outlined the data collection process in full detail. For example, it is possible that the proxy also assisted the person with dementia with their self-report, which could have resulted in biased estimates and the need for future studies applying blinding. Although we assessed the risk of bias of included studies, the checklist was not directly reflecting the purpose of our study that looked into inter-rater agreement. No checklist for this purpose currently exists. Finally, quality appraisal by a second reviewer was only conducted for the first 25% of the studies due to resource constraints and a low rate of disagreement between the two assessors. However, an agreement index between reviewers regarding the concordance in selecting full texts for inclusion and conducting risk of bias assessments was not calculated.

## Conclusion

This review demonstrates that the choice of proxy perspective impacts the inter-rater gap. QoL scores from the proxy-patient perspective align more closely with self-reported scores than the proxy-proxy perspective. These findings contribute to the broader literature investigating factors influencing differences in QoL scores between proxies and individuals with dementia. While self-reported QoL is the gold standard, proxy-reports should be viewed as complements rather than substitutes. Both proxy perspectives offer unique insights, yet QoL assessments in people with dementia are complex. The difference in self- and proxy-reports can be influenced by various factors, necessitating further research before presenting definitive results that inform care provision and policy.

### Supplementary Information

Below is the link to the electronic supplementary material.

Supplementary file1 (XLSX 67 KB)

Supplementary file2 (DOCX 234 KB)

## Data Availability

All data associated with the systematic literature review are available in the supplementary file.
