# Supplementary material for: Understanding the influence of different proxy perspectives in explaining the difference between self-rated and proxy-rated quality of life in people living with dementia: a systematic literature review and meta-analysis
Source: Qual Life Res. 2024 Apr 24;33(8):2055–66. doi: 10.1007/s11136-024-03660-w (PMC11286712; doi:10.1007/s11136-024-03660-w)
Supplement: Supplementary file 2 — Supplementary file2 (DOCX 234 KB) [file 11136_2024_3660_MOESM2_ESM.docx]

**SUPPLEMENTAL MATERIAL**

**Understanding the influence of different proxy perspectives in explaining the difference between self-rated and proxy-rated quality of life in people living with dementia: a systematic literature review**

**Authors:**

Lidia Engel,^1^

Valeriia Sokolova,^1^

Ekaterina Bogatyreva,^2^

Anna Leuenberger ^2^

**Affiliations:**

1. School of Public Health and Preventive Medicine, Monash University, Melbourne, Victoria, Australia.
2. School of Health and Social Development, Deakin University, Burwood, Victoria, Australia.

***Corresponding author:**

Lidia Engel, PhD

School of Public Health and Preventive Medicine, Monash University, Level 4, 553 St. Kilda Road, Melbourne, VIC 3004 Australia

Email: [lidia.engel@monash.edu](mailto:lidia.engel@monash.edu). Phone: +61 3 9903 8961

**Elements included in the online only supplemental material:**

**Appendix 1: PRISMA checklist**

**Appendix 3: Search strategy in MEDLINE**

**Appendix 4: Characteristics of studies**

**Appendix 4: Quality assessment scores**

**Appendix 5: Pooled raw mean difference by proxy perspective and QOL domains**

**Appendix 1: PRISMA 2020 checklist**

| **Section and Topic** | **Item #** | **Checklist item** | **Location where item is reported** |
| --- | --- | --- | --- |
| **TITLE** | | |  |
| Title | 1 | Identify the report as a systematic review. | Title |
| **ABSTRACT** | | |  |
| Abstract | 2 | See the PRISMA 2020 for Abstracts checklist. | Abstract |
| **INTRODUCTION** | | |  |
| Rationale | 3 | Describe the rationale for the review in the context of existing knowledge. | Introduction |
| Objectives | 4 | Provide an explicit statement of the objective(s) or question(s) the review addresses. | Introduction |
| **METHODS** | | |  |
| Eligibility criteria | 5 | Specify the inclusion and exclusion criteria for the review and how studies were grouped for the syntheses. | Inclusion criteria |
| Information sources | 6 | Specify all databases, registers, websites, organisations, reference lists and other sources searched or consulted to identify studies. Specify the date when each source was last searched or consulted. | Search strategy |
| Search strategy | 7 | Present the full search strategies for all databases, registers and websites, including any filters and limits used. | Appendix 1 |
| Selection process | 8 | Specify the methods used to decide whether a study met the inclusion criteria of the review, including how many reviewers screened each record and each report retrieved, whether they worked independently, and if applicable, details of automation tools used in the process. | Screening |
| Data collection process | 9 | Specify the methods used to collect data from reports, including how many reviewers collected data from each report, whether they worked independently, any processes for obtaining or confirming data from study investigators, and if applicable, details of automation tools used in the process. | Data extraction |
| Data items | 10a | List and define all outcomes for which data were sought. Specify whether all results that were compatible with each outcome domain in each study were sought (e.g. for all measures, time points, analyses), and if not, the methods used to decide which results to collect. | Data extraction |
|  | 10b | List and define all other variables for which data were sought (e.g. participant and intervention characteristics, funding sources). Describe any assumptions made about any missing or unclear information. | Data extraction |
| Study risk of bias assessment | 11 | Specify the methods used to assess risk of bias in the included studies, including details of the tool(s) used, how many reviewers assessed each study and whether they worked independently, and if applicable, details of automation tools used in the process. | Risk of bias and quality assessment |
| Effect measures | 12 | Specify for each outcome the effect measure(s) (e.g. risk ratio, mean difference) used in the synthesis or presentation of results. | Data extraction |
| Synthesis methods | 13a | Describe the processes used to decide which studies were eligible for each synthesis (e.g. tabulating the study intervention characteristics and comparing against the planned groups for each synthesis (item #5)). | Data synthesis and analysis |
|  | 13b | Describe any methods required to prepare the data for presentation or synthesis, such as handling of missing summary statistics, or data conversions. | Data synthesis and analysis |
|  | 13c | Describe any methods used to tabulate or visually display results of individual studies and syntheses. | Data synthesis and analysis |
|  | 13d | Describe any methods used to synthesize results and provide a rationale for the choice(s). If meta-analysis was performed, describe the model(s), method(s) to identify the presence and extent of statistical heterogeneity, and software package(s) used. | Data synthesis and analysis |
|  | 13e | Describe any methods used to explore possible causes of heterogeneity among study results (e.g. subgroup analysis, meta-regression). | Data synthesis and analysis |
|  | 13f | Describe any sensitivity analyses conducted to assess robustness of the synthesized results. | Data synthesis and analysis |
| Reporting bias assessment | 14 | Describe any methods used to assess risk of bias due to missing results in a synthesis (arising from reporting biases). | Data synthesis and analysis |
| Certainty assessment | 15 | Describe any methods used to assess certainty (or confidence) in the body of evidence for an outcome. | Data synthesis and analysis |
| **RESULTS** | | |  |
| Study selection | 16a | Describe the results of the search and selection process, from the number of records identified in the search to the number of studies included in the review, ideally using a flow diagram. | General study characteristics |
|  | 16b | Cite studies that might appear to meet the inclusion criteria, but which were excluded, and explain why they were excluded. | N/A |
| Study characteristics | 17 | Cite each included study and present its characteristics. | Appendix 2; General study characteristics |
| Risk of bias in studies | 18 | Present assessments of risk of bias for each included study. | Appendix 3 |
| Results of individual studies | 19 | For all outcomes, present, for each study: (a) summary statistics for each group (where appropriate) and (b) an effect estimate and its precision (e.g. confidence/credible interval), ideally using structured tables or plots. | Supplementary file2; Mean scores and mean differences by proxy perspective and QoL measure |
| Results of syntheses | 20a | For each synthesis, briefly summarise the characteristics and risk of bias among contributing studies. | Results |
|  | 20b | Present results of all statistical syntheses conducted. If meta-analysis was done, present for each the summary estimate and its precision (e.g. confidence/credible interval) and measures of statistical heterogeneity. If comparing groups, describe the direction of the effect. | Standardized mean differences by proxy perspective, stratified by proxy type, dementia severity and living arrangement; Inter-rater agreement (ICC) statistics |
|  | 20c | Present results of all investigations of possible causes of heterogeneity among study results. | Results |
|  | 20d | Present results of all sensitivity analyses conducted to assess the robustness of the synthesized results. | N/A |
| Reporting biases | 21 | Present assessments of risk of bias due to missing results (arising from reporting biases) for each synthesis assessed. | Mean scores and mean differences by proxy perspective and QoL measure |
| Certainty of evidence | 22 | Present assessments of certainty (or confidence) in the body of evidence for each outcome assessed. | Results |
| **DISCUSSION** | | |  |
| Discussion | 23a | Provide a general interpretation of the results in the context of other evidence. | Discussion |
|  | 23b | Discuss any limitations of the evidence included in the review. | Limitations and recommendations for future studies |
|  | 23c | Discuss any limitations of the review processes used. | Limitations and recommendations for future studies |
|  | 23d | Discuss implications of the results for practice, policy, and future research. | Limitations and recommendations for future studies |
| **OTHER INFORMATION** | | |  |
| Registration and protocol | 24a | Provide registration information for the review, including register name and registration number, or state that the review was not registered. | N/A |
|  | 24b | Indicate where the review protocol can be accessed, or state that a protocol was not prepared. | N/A |
|  | 24c | Describe and explain any amendments to information provided at registration or in the protocol. | N/A |
| Support | 25 | Describe sources of financial or non-financial support for the review, and the role of the funders or sponsors in the review. | Funding |
| Competing interests | 26 | Declare any competing interests of review authors. | Conflict of interest |
| Availability of data, code and other materials | 27 | Report which of the following are publicly available and where they can be found: template data collection forms; data extracted from included studies; data used for all analyses; analytic code; any other materials used in the review. |  |

*From:*  Page MJ, McKenzie JE, Bossuyt PM, Boutron I, Hoffmann TC, Mulrow CD, et al. The PRISMA 2020 statement: an updated guideline for reporting systematic reviews. BMJ 2021;372:n71. doi: 10.1136/bmj.n71

**Appendix 2: Search strategy in Medline (EBSCOhost) performed on 9 December 2021**

| **#** | **Query** | **Results** |
| --- | --- | --- |
| S69 | Limiters- Date of Publication: 20180101-20211231; English Language; Human | 3,394 |
| S68 | Limiters- Date of Publication: 20180101-20221231; English Language | 4,381 |
| S67 | Limiters- Date of Publication: 20180101-20221231 | 4,475 |
| S66 | S65 NOT S64 | 13,533 |
| S65 | S17 AND S34 AND S49 | 14,649 |
| S64 | S50 OR S51 OR S52 ORS53 OR S54 OR S55 ORS56 OR S57 OR S58 ORS59 OR S60 OR S61 ORS62 OR S63 | 4,315,455 |
| S63 | AB paediatric* OR TI paediatric* | 71,472 |
| S62 | AB pediatric* OR TI pediatric* | 328,606 |
| S61 | AB adolescent* OR TI adolescent* | 281,723 |
| S60 | AB infant* OR TI infant* | 424,485 |
| S59 | AB "child* health" OR TI "child* health" | 31,381 |
| S58 | AB "child* care*" OR TI "child* care*" | 9,291 |
| S57 | AB child* OR TI child* | 1,493,257 |
| S56 | (MH "Pediatrics+") | 61,589 |
| S55 | (MH "Adolescent Health") | 1,625 |
| S54 | (MH "Adolescent") | 2,141,666 |
| S53 | (MH "Infant+") | 1,197,777 |
| S52 | (MH "Child Health") | 4,042 |
| S51 | (MH "Child Care+") | 20,939 |
| S50 | (MH "Child+") | 2,030,315 |
| S49 | S35 OR S36 OR S37 OR S38 OR S39 OR S40 OR S41 OR S42 OR S43 OR S44 OR S45 OR S46 OR S47 OR S48 | 617,544 |
| S48 | AB "lewy bod*" OR TI "lewy bod*" | 10,268 |
| S47 | AB "cognitive disease*"OR TI "cognitive disease*" | 179 |
| S46 | AB "cognitive deficit*"OR TI "cognitive deficit*" | 23,383 |
| S45 | AB neurodegen* OR TI neurodegen* | 125,170 |
| S44 | AB "memor* disorder*"OR TI "memor* disorder*" | 1,843 |
| S43 | AB "cognitiv* impairment*" OR TI "cognitiv* impairment*" | 74,421 |
| S42 | AB "cognitive dysfunction*" OR TI "cognitive dysfunction*" | 16,866 |
| S41 | AB alzheimer* OR TI alzheimer* | 161,859 |
| S40 | AB dementia* OR TI dementia* | 122,185 |
| S39 | (MH "Neurodegenerative Diseases+") | 332,973 |
| S38 | (MH "Memory Disorders+") | 31,300 |
| S37 | (MH "Cognitive Dysfunction+") | 26,683 |
| S36 | (MH "Alzheimer Disease") | 104,603 |
| S35 | (MH "Dementia+") | 183,547 |
| S34 | S18 OR S19 OR S20 ORS21 OR S22 OR S23 ORS24 OR S25 OR S26 ORS27 OR S28 OR S29 ORS30 OR S31 OR S32 ORS33 | 3,148,560 |
| S33 | AB "outcome*assessment*" OR TI "outcome* assessment*" | 7,133 |
| S32 | AB (((social or physical*or emotional* or cognitive*) N2 function*)) OR TI (((social or physical* or emotional*or cognitive*) N2function*)) | 145,918 |
| S31 | AB (((treatment* or care* or disease*) N2experience*)) OR TI (((treatment* or care* or disease*) N2experience*)) | 60,473 |
| S30 | AB "well being" OR TI "well being" | 90,231 |
| S29 | AB assessment* OR TI assessment* | 1,186,644 |
| S28 | AB instrument* OR TI instrument* | 307,787 |
| S27 | AB "patient* status" OR TI "patient* status" | 3,083 |
| S26 | AB "health status" OR TI "health status" | 63,881 |
| S25 | AB "symptom*assessment*" OR TI "symptom* assessment*" | 3,828 |
| S24 | AB HRQL OR TI HRQL | 3,760 |
| S23 | AB HRQOL OR TI HRQOL | 19,039 |
| S22 | AB "quality of life" OR TI "quality of life" | 317,384 |
| S21 | (MH "Outcome Assessment, HealthCare+") | 1,246,464 |
| S20 | (MH "Patient Outcome Assessment+") | 16,664 |
| S19 | (MH "Health Status+") | 387,818 |
| S18 | (MH "Quality of Life") | 228,071 |
| S17 | S1 OR S2 OR S3 OR S4OR S5 OR S6 OR S7OR S8 OR S9 OR S10OR S11 OR S12 OR S13OR S14 OR S15 OR S16 | 373,520 |
| S16 | AB care* N2 report* OR TI care* N2 report* | 22,000 |
| S15 | AB "external* rate*" OR TI "external* rate*" | 127 |
| S14 | AB "self assessment*"OR TI "self assessment*" | 13,476 |
| S13 | AB "external* rating" OR TI "external* rating" | 18 |
| S12 | AB "patient* agent*" OR TI "patient* agent*" | 88 |
| S11 | AB caregiver* OR TI caregiver* | 75,068 |
| S10 | AB carer* OR TI carer* | 16,349 |
| S9 | AB "informal* care*" ORTI "informal* care*" | 6,312 |
| S8 | AB "self report*" OR TI "self report*" | 177,899 |
| S7 | AB "informal* care*" N2report* OR TI "informal*care*" N2 report* | 147 |
| S6 | AB (((care* or family or families or "next of kin" or spouse* or husband* or wife* or partner* or relative* or clinician* or physician* or nurse* or doctor* or "health professional*" or "medical professional*")N2 report*)) OR TI (((care* or family or families or "next of kin" or spouse* or husband* or wife* or partner* or relative* or clinician* or physician* or nurse* or doctor* or "health professional*" or "medical professional*")N2 report*)) | 72,933 |
| S5 | AB "health care agent*"OR TI "health care agent*" | 59 |
| S4 | AB proxies OR TI proxies | 31,001 |
| S3 | AB proxy OR TI proxy | 31,001 |
| S2 | (MH "Caregivers") | 43,385 |
| S1 | (MH "Proxy") | 1,749 |

**Appendix 3: Characteristics of studies**

| **Author (year), country** | **Study design (cross-sectional, RCT etc)** | **Study sample, total dyads** | **Setting (community, residential, mixed, undefined)** | **Proxy type (formal, informal, mixed, undefined)** | **Dementia type (AD, Lewy body, Vascular, mixed etc.)** | **Dementia severity (mild, moderate, severe)** | **MMSE mean score, SD** |
| --- | --- | --- | --- | --- | --- | --- | --- |
| Aguirre (2016), UK [1] | Cross-sectional study | N=272 | Mixed | Informal | Any | Mild-to-moderate dementia on CDR | 16.75 (5.53) |
| Aleixo (2022), Brazil [2] | RCT | N=13 | Community | Mixed | AD (10),  VaD (2), MD (1) | Mild (CDR) -1)  Moderate (CDR-2) | 16.38 (SD: 2.87) |
| Andrieu (2016), France [3] | Cohort study | N=574 | Community | Informal | AD | Mild to moderate (MMSE score between 12 and 26) | 19.5 (3.9) |
| Arons (2013), Netherlands [4] | Cohort study | N=175 | Community | Informal | Any | Mild to moderate (DSM-IV-TR criteria and a score of 0.5-2 on the CDR scale 0–3 | 22.8 (3.4) |
| Ascher-Svanum (2015), USA [5] | Cross-sectional study | N=972 | Community | Informal | AD | Mild (MMSE 20-26) | 22.9 (2.0) |
| Baptista (2019), Brazil [6] | Cross-sectional study | N=132 | Community | Informal | AD or vascular | Mild and moderate (MMSE  11 to 26) | 18.31 |
| Barbe (2018), France & Switzerland [7] | Cross-sectional study | N=123 | Mixed | Informal | AD | Mild to moderate (DSM-IV-TR) | 20.7 (4.5) |
| Barrios (2013), Portugal [8] | Cross-sectional study | N=104 | Community | Informal | AD or Vascular | Mild cognitive impairment (European Consortium on Alzheimer’s Disease criteria) or mild-to-moderate dementia (NINCDS-ADRDA criteria or NINDS-AIREN criteria) | 21.3 (8.8) |
| Beerens (2014), EU [9] | Cross-sectional study | N=1347 | Mixed | Informal | Any | (S-MMSE 24 or lower) | - |
| Belfort (2020), Brazil [10] | Cross-sectional study | N=137 | Residential | Informal | AD | Mild to moderate (MMSE 12-26) | 18.7 (4.4) |
| Bosboom (2012), Australia [11] | Cross-sectional study | N=80 | Community | Informal | AD | Mild or moderate severity (NINCDS-ADRD criteria; MMSE ≥10) | - |
| Bosboom (2014), Australia [12] | Cross-sectional study | N=47 | Community | Informal | AD | Mild or moderate severity (NINCDS-ADRD criteria; MMSE ≥10) | - |
| Bostrom (2007), Sweden [13] | Cross-sectional study | N=68 | Mixed | Informal | AD and Lewy body | DLB criteria and ICD-10 criteria for AD | DLB patients: 17.3 (0-29) AD patients: 16.9 (0-30) |
| Brennan (2023), Japan [14] | RCT | N=101 | Residential | Formal | unknown (39), AD (33), VaD (5), Parkinson’s associated dementia (3), Lewy body dementia (1), MD (1), developing dementia (19) | Undefined | mean FAST stage = 5.1 (SD: 0.8, range stage 3–6) equivalent of MMSE = 10-15 |
| Bruvik (2012), Norway [15] | RCT | N=230 | Community | Informal | Not defined | the ICD-10 criteria, MMSE score of at least 15 points | 21.3 (3.6) |
| Crespo (2013), Spain [16] | Cross-sectional study | N=209 | Residential | Informal | Not defined | MMSE score of less than 27 | 13.3 (5.88) |
| Cummings (2006), USA [17] | Cross-sectional study | N=644 | Community | Informal | MCI and AD | mMMSE score 88 or higher for subjects with more than 8 years of education;  mMMSE score 80 or higher for subjects with less than 8 years of education | mMMSE 95 (3.7) |
| D'Cunha (2019), Australia [18] | Cross-sectional study | N=25 | Residential | Mixed | AD (17), vascular (3), Parkinson’s (2), mixed (3) | Dementia diagnosis (any form) | - |
| Dewitte (2017), Belgium [19] | Cross-sectional study | N=88 | Residential | Formal | AD | All stages (no MMSE limit) | 15.99 (5.73) |
| Dixit (2021), UK [20] | Cross-sectional study | N=26 | Community | Informal | 17 (65%) AD, 5 (19%) FTD, and 5 (19%) other dementia subtypes | - | - |
| Dourado (2021), Brazil [21] | Cross-sectional study | N=256 | Community | Informal | AD | Mild to moderate AD according to the CDR scale and MMSE scores of 14 to 26 | 19.76 (3.89) |
| Edelman (2004), US [22] | Cross-sectional study | N=54 | Community | Formal | Not defined | Mild to moderate dementia as defined by an MMSE score of 10 or greater | 12.5 (7.3) |
| Edelman (2005), US [23] | Cross-sectional study | N=65 | Residential | Formal | Not defined | Mild to moderate (MMSE scores 10 or higher) | 9.0 (6.9) |
| Farina (2020), UK [24] | Cross-sectional study | N=210 | Mixed | Informal | AD (118), VaD (25), LBD (3), FTD (8), other (41) | mild (sMMSE), moderate (sMMSE 10–19) | Mild sMMSE 24.6 (0.2)  Moderate sMMSE 15.1 (2.9) |
| Farina (2022), UK [25] | Cohort study | N=87 | Community | Informal | AD (47) | Mild  Moderate Severe | sMMSE 21.21 (5.26) |
| Felekoglu (2021), Turkey [26] | Cross-sectional study | N=73 | Community | Informal | AD | Mild to moderate (at least 10 points on the MMSE) | 19.38 (4.53) |
| Ferry (2020), UK and Northern Ireland [27] | Cost-effectiveness analysis (alongside a trial) | N=60 | Community | Informal | Not defined | Mild to moderate | - |
| Fleming (2014), Australia [28] | Cross-sectional study | N=275 | Residential | Formal | Any | Not defined | - |
| Froelich (2021), Germany, Spain, UK [29] | Cohort study | N=616 | Residential | Informal | AD | Mild to moderate (mild AD: MMSE 21–26 points; moderate AD: MMSE 10–20 points) | 19.40 (4.09) |
| Fuh (2006), Taiwan [30] | Cross-sectional study | N=81 | Community | Informal | AD | Mild to moderate (DSM-IV criteria) | 20.1 (4.5) |
| Gibbor (2021), UK [31] | RCT | N=33 | Residential | Informal | Not defined | Mild to moderate (at least 10/30 on SMMSE) | SMMSE 21.70 (3.51) |
| Giebel (2014), UK [32] | Cross-sectional study | N=1223 | Mixed | Informal | Any | Mild moderate or severe (a score of 24 or less on SMMSE) | Mild 21.8 (1.4)  Moderate 15 (2.9)  Severe 4.8 (3.5) |
| Giebel (2015), UK [33] | Cross-sectional study | N=122 | Mixed | Informal | Any | Mild moderate or severe (a score of 24 or less on SMMSE) | Mild 22 (1.3)  Moderate 14 (2.5)  Severe 4.1 (3) |
| Giebel (2015), EU [34] | Cross-sectional study | N=414 | Mixed | Mixed | Any | Severe (SMMSE score of 24 or below) | SMMSE 4.9 (3.1) |
| Gomez-Gallego (2012), Spain [35] | Cross-sectional study | N=102 | Community | Informal | AD | Mild moderate and severe (NINCDS-ADRDA criteria) | - |
| Gomez-Gallego (2015), Spain [36] | Cross-sectional study | N=276 | Community | Informal | AD | Mild to moderate (4 or 5 in the Global Deterioration Scale score) | 18.51 (4.29) |
| Gräske (2012), Germany [37] | Cross-sectional study | N=49 | Residential | Formal | Any | Mild moderate severe (Global Deterioration Scale) | 17.6 (7.9) |
| Handels (2018), EU [38] | Cross-sectional study | N=451 | Community | Informal | Any | Mild to moderate (MMSE ≤24) | 19.0 (5.0) |
| Heggie (2012), Canada [39] | Cohort study | N=119 | Community | Informal | Any | Mild to moderate | 3MS 70.6 (18.7) |
| Hessmann (2018), Germany [40] | Cross-sectional study | N=395 | Mixed | Informal | AD | Mild moderate severe (clinical diagnostic criteria (NINCDS-ADRDA) | 14.3 (9.2) |
| Hilgeman (2014), US [41] | Cross-sectional study | N=18 | Community | Informal | Any | Mild (CDR 0.5 very mild or questionable dementia or 1 mild dementia) | - |
| Huang (2009), Taiwan [42] | Cross-sectional study | N=120 | Community | Informal | AD or related dementia | Mild moderate to severe | 18.64 (6.23) |
| Inouye (2010), Brazil [43] | Cross-sectional study | N=53 | Community | Informal | AD | Mild to moderate (MMSE and CDR) | - |
| Jayakody (2023), Sri Lanka [44] | Cross-sectional study | N=272 | Residential | Mixed | Any | Mild (DSM-IV classification)  Moderate (DSM-IV classification) | Not defined |
| Jönsson (2006), Sweden, Denmark [45] | Cohort study | N=376 | Mixed | Informal | AD | Mild moderate or severe | 19.2 |
| Kimura (2018), Brazil [46] | Cross-sectional study | N=110 | Community | Informal | AD | Mild to moderate (DSM-IV-TR) | 20.6 (4.0)  20.0 (3.5) |
| Kimura (2021), Brazil [47] | Cross-sectional study | N=110 | Community | Informal | AD | Mild to severe (MMSE 12 to 26) | 16.92 (6.0) |
| Kisvetrova (2018), Czech Republic [48] | Cross-sectional study | N=212 | Community | Informal | Any | Mild | 22.6 (1.7) |
| Kunz (2010), Germany [49] | RCT | N=333 | Community | Informal | Any | Mild to moderate (MMSE between 10 and 24 points0 | 18.6 (3.8) |
| Lacerda (2020), Brazil [50] | Cross-sectional study | N=128 | Community | Informal | AD | Mild to moderate (11 to 26 in MMSE) | Mild: 21.11 (3.112) Moderate: 14.74 (2.316) |
| Lacey (2015), USA and Canada [51] | RCT | N=2204 | Community | Informal | AD | Mild to moderate (MMSE of 16 to 26) | 21.0 (3.22) |
| Lamb (2018), UK [52] | RCT | N=494 | Community | Informal | Any | Mild to moderate (a score of > 10 on the sMMSE) | sMMSE  21.6 (4.6)  22.0 (4.7) |
| Lee (2023), Australia [53] | Cross-sectional study | N=26 | Community | Informal | Any (AD 10, mixed 2) | mild dementia (RUDAS score 17–22), moderate (RUDAS score 10–16) and severe dementia (RUDAS score <10) | RUDAS 16.9 (4.7) |
| Leontjevas (2016), Netherlands [54] | Cross-sectional study | N=582 | Residential | Formal | Any | - | 16.45 (5.10) |
| Leroi (2019), France, UK, Cyprus [55] | RCT | N=19 | Community | Informal | AD, mixed, Vascular dementia | Mild to moderate (MoCA score of ≥12) | - |
| Logsdon (1999), US [56] | Cross-sectional study | N=77 | Community | Informal | AD | Mild to moderate (NINCDS-ADRDA) | 17.1 (5.6) |
| Matsui (2006), Japan [57] | Cross-sectional study | N=140 | Community | Informal | AD | Mild to moderate (MMSE higher than 10) | 20.3 (4.2) |
| Moon (2016), USA [58] | Cross-sectional study | N=200 | Community | Informal | Any | Mild to moderate (MMSE score between 13 and 26) | - |
| Moyle (2012), Australia [59] | Cross-sectional study | N=58 | Residential | Formal | AD | Mild to moderate (MMSE of 12–24) | - |
| Naglie (2006), Canada [60] | Cross-sectional study | N=60 | Mixed | Informal | AD | Mild dementia: MMSE 19– 26; moderate dementia: MMSE 10–18 | 18.9 (4.5) |
| Niikawa (2019), Japan [61] | Cross-sectional study | N=87 | Mixed | Informal | Any | Mild-to-moderate dementia (MMSE ≥10) | 19.28 (3.60) |
| Nogueira (2021), Brazil [62] | Cross-sectional study | N=98 | Community | Informal | AD | Mild to moderate (MMSE scores of 13 to 26 and classified CDR 1 (mild) or 2 (moderate)) | 20.4 (4.6)  18.8 (3.7) |
| Novelli (2010), Brazil [63] | Cross-sectional study | N=60 | Mixed | Informal | AD | Mild-to-moderate (DSM-III-R criteria and MMSE 10 to 25) | 20.2 (2.6)  14.0 (2.8) |
| O'Shea (2020), EU [64] | Cross-sectional study | N=451 | Community | Informal | Any | Mild to moderate dementia (according to DSM-IV-TR criteria) | 19 (4.99) |
| Olthof-Nefkens (2023), Netherlands [65] | Cohort study | N=57 | Community | Undefined | AD (50), Lewy body (1), Frontotemporal dementia (1), Primary progressive aphasia (1), mixed (4) | Mild to moderate dementia (CDR score between 0.5 and 2) | 21.8 (7–29) ± 4.4 |
| Orgeta (2015), UK [66] | Cross-sectional study | N=488 | Community | Informal | Any | Mild to moderate (DSM-IV criteria) | - |
| Phung (2018), Denmark [67] | Cross-sectional study | N=330 | Community | Informal | AD | Mild (MMSE score ≥ 20) | 24.1 (2.6) |
| Pizzo (2023), UK [68] | Cost-utility analysis (alongside a trial) | N=468 | Community | Informal | Any | Mild to moderate (the DSM-IV; and score between 0.5 and 2 on the CDR Scale) | - |
| Polat (2022), Turkey [69] | RCT | N=11 | Community | Undefined | AD | Mild (CDR: 1) | 24.27 (SD: 3.31) |
| Ready (2004), US [70] | Cross-sectional study | N=79 | Community | Informal | AD | Mild (CDR rating of 0.5 or 1) | 28.9 (1.3)  27.4 (2.0)  22.9 (3.8) |
| Rokstad (2017), Norway [71] | Cross-sectional study | N=261 | Community | Informal | AD (206), VD (22), mixed (14), LBD (13), other (6) | Mild and moderate (MMSE score of ≥15) | 20.4 (3.6)  20.3 (3.6) |
| Rombach (2020), EU [72] | Cross-sectional study | N=1020 | Community | Informal | Any | Mild moderate (MMSE score of 24 or below) | 19 (5)  25 (4) |
| Romhild (2018), EU [73] | Cross-sectional study | N=1330 | Mixed | Mixed | Any | Mild to moderate (SMMSE 24 and lower) | SMMSE  15.0 (5.7) |
| Ruggero (2023), Australia [74] | Cross-sectional study | N=18 | Community | Informal | Primary Progressive Aphasia | a formal diagnosis of PPA | - |
| Samus (2014), US [75] | RCT | N=303 | Community | Informal | Any | Mild moderate and severe (TICS <31 and IQCODE >52 cutoffs) | 19.1 (7.8) |
| Sands (2004), US [76] | Cross-sectional study | N=91 | Community | Informal | Any | Mild to moderate (MMSE 12 or greater) | 19.7 (4.5) |
| Santos (2022), Brazil [77] | Cross-sectional study | N=102 | Community | Informal | AD | Mild and moderate (11-26 scores on MMSE) | 19.2 (4.2) |
| Sari (2023), Indonesia [78] | RCT | N=30 | Community | Informal | Any (AD 22, vascular 6, Frontotemporal 1, Parkinson’s dementia 1) | Mild to moderate severity (Telephone MMSE (T-MMSE) score of 8–23) | T-MMSE 15.0 (3.3) |
| Schumann (2019), Germany [79] | Cohort study | N=100 | Mixed | Informal | Any | Mild moderate and severe (CDR; range: 0‐3) | 17.83 (7.02) |
| Sheehan (2012), UK [80] | Cross-sectional study | N=109 | Residential | Informal | Any | Mild moderate and severe (CDR; range: 0‐3) | - |
| Shikimoto (2020), Japan [81] | Cross-sectional study | N=77 | Community | Informal | AD | Mild to moderate (mild: MMSE score of less than 26 and more than 20 points; moderate: a score of less than 19 and more than 15 points) | - |
| Snow (2005), USA [82] | Cross-sectional study | N=89 | Community | Informal | Any | Not defined | - |
| Sousa (2013), Brazil [83] | Cohort study | N=41 | Community | Informal | AD | Mild (MMSE scores of 13–26) | 21.0 (3.93) |
| Sousa (2018), Brazil [84] | Cross-sectional study | N=75 | Community | Informal | AD | Mild (CDR=1 and MMSE scores of 13 to 26) | 21.2 (3.5) |
| Söylemez (2020), Turkey [85] | Cross-sectional study | N=98 | Community | Informal | AD | mild to moderate (MMSE 10 and higher) | 17.38 (4.31) |
| Tay (2014), Singapore [86] | Cross-sectional study | N=165 | Community | Informal | Any | Mild to moderate (CDR global score of>0 and ࣘ2) | CMMSE  18.4 (4.2) |
| Torisson (2016), Sweden [87] | Cross-sectional study | N=139 | Community | Informal | Any | Not defined | 22.6 (4.5) |
| Trigg (2015), UK [88] | Cohort study | N=145 | Mixed | Informal | AD | Mild, moderate or severe | 14.99 (7.0) |
| van Santen (2020), Netherlands [89] | RCT | N=112 | Community | Informal | Any | Not defined | 18.1 (6.7)  19.4 (6.5) |
| Wolak (2009), France [90] | Cross-sectional study | N=120 | Mixed | Informal | AD | Mild to moderate (a score of 10 on the MMSE) | 20.8 (4.5) |
| Wu (2020), UK [91] | Cohort study | N=1283 | Community | Informal | AD (715), VaD (142), mixed (263), FTD (45), PDD (43), DLB (43), other (32) | Mild to moderate (MMSE score≥15) | - |
| Yamada (2020), Japan [92] | Cross-sectional study | N=132 | Community | Informal | AD | Mild to moderate (MMSE score≥10) | 20.2 (4.2) |
| Yeaman (2012), US [93] | Cross-sectional study | N=10 | Community | Informal | AD | Mild to moderate (MMSE score≥10) | 17.80 |
| Yu (2013), China [94] | Cross-sectional study | N=87 | Community | Informal | AD | Not defined | - |
| Zhao (2011), France [95] | Cross-sectional study | N=122 | Mixed | Informal | AD | Mild to moderate (MMSE score≥10) | 21 (5.0) |
| Zucchella (2015), Italy [96] | Cross-sectional study | N=135 | Community | Informal | AD | Mild to moderate (NINCDS-ADRDA) | 15.3 (7.5) |

AD= Alzheimer’s Disease, CDR=Clinical Dementia Rating Scale; DSM-IV-TR=Diagnostic and Statistical Manual of Mental Disorders, 4th Edition, Text Revision; MD= Mixed dementia; MMSE=Mini-Mental State Examination; MoCA= Montreal Cognitive Assessment; RCT – randomised controlled trial; RUDAS= Rowland Universal Dementia Assessment Scale; VaD=Vascular Dementia.

**Appendix 4: Quality assessment scores**

|  | **Q1** | **Q2** | **Q3** | **Q4** | **Q5** | **Q6** | **Q7** | **Q8** | **Q9** | **Q10** | **Q11** | **Q12** | **Q13** | **Q14** | **Quality score (%)** |
| --- | --- | --- | --- | --- | --- | --- | --- | --- | --- | --- | --- | --- | --- | --- | --- |
| Aguirre (2016) | 2 | 1 | 2 | 2 | n/a | n/a | n/a | 2 | n/a | 2 | 1 | n/a | 2 | 2 | 89 |
| Aleixo (2022) | 2 | 1 | 1 | 2 | n/a | n/a | n/a | 2 | 0 | 2 | 1 | n/a | 2 | 1 | 70 |
| Andrieu (2016) | 2 | 2 | 2 | 2 | n/a | n/a | n/a | 2 | 2 | 2 | 2 | n/a | 2 | 2 | 100 |
| Arons (2013) | 1 | 1 | 1 | 2 | n/a | n/a | n/a | 2 | 2 | 2 | 2 | n/a | 2 | 2 | 85 |
| Ascher-Svanum (2015) | 2 | 2 | 2 | 2 | n/a | n/a | n/a | 2 | 2 | 2 | 2 | n/a | 2 | 2 | 100 |
| Baptista (2019) | 2 | 2 | 2 | 2 | n/a | n/a | n/a | 2 | 1 | 2 | 2 | n/a | 2 | 2 | 95 |
| Barbe (2018) | 2 | 2 | 2 | 2 | n/a | n/a | n/a | 2 | n/a | 2 | 1 | n/a | 2 | 2 | 94 |
| Barrios (2013) | 2 | 1 | 2 | 2 | n/a | n/a | n/a | 2 | 1 | 2 | 1 | 1 | 1 | 1 | 73 |
| Beerens (2014) | 2 | 2 | 2 | 2 | n/a | n/a | n/a | 2 | n/a | 2 | 2 | n/a | 2 | 2 | 100 |
| Belfort (2020) | 2 | 1 | 2 | 2 | n/a | n/a | n/a | 2 | n/a | 2 | 1 | n/a | 2 | 2 | 89 |
| Bosboom (2012) | 2 | 2 | 2 | 2 | n/a | n/a | n/a | 2 | n/a | 2 | 2 | n/a | 2 | 2 | 100 |
| Bosboom (2014) | 1 | 2 | 2 | 2 | n/a | n/a | n/a | 2 | n/a | 2 | 2 | n/a | 2 | 2 | 94 |
| Bostrom (2007) | 2 | 2 | 2 | 2 | n/a | n/a | n/a | 2 | 2 | 2 | 2 | 2 | 2 | 2 | 100 |
| Brennan (2023) | 2 | 2 | 2 | 1 | n/a | n/a | n/a | 2 | 2 | 2 | 2 | n/a | 2 | 2 | 95 |
| Bruvik (2012) | 2 | 2 | 2 | 2 | n/a | n/a | n/a | 2 | 2 | 2 | 1 | 2 | 2 | 2 | 95 |
| Crespo (2013) | 2 | 2 | 2 | 1 | n/a | n/a | n/a | 2 | n/a | 2 | 2 | n/a | 2 | 2 | 94 |
| Cummings (2006) | 1 | 1 | 2 | 2 | n/a | n/a | n/a | 2 | n/a | 2 | 1 | n/a | 2 | 2 | 83 |
| D'Cunha (2019) | 2 | 2 | 2 | 2 | n/a | n/a | n/a | 2 | n/a | 2 | 1 | n/a | 2 | 2 | 94 |
| Dewitte (2018) | 1 | 1 | 2 | 2 | n/a | n/a | n/a | 2 | n/a | 2 | 1 | n/a | 2 | 2 | 83 |
| Dixit (2021) | 2 | 2 | 2 | 1 | n/a | n/a | n/a | 2 | n/a | 2 | 1 | n/a | 2 | 2 | 89 |
| Dourado (2021) | 2 | 2 | 2 | 2 | n/a | n/a | n/a | 2 | n/a | 2 | 2 | n/a | 2 | 2 | 100 |
| Edelman (2004) | 1 | 2 | 2 | 1 | n/a | n/a | n/a | 2 | n/a | 2 | 1 | n/a | 2 | 1 | 78 |
| Edelman (2005) | 1 | 2 | 2 | 2 | n/a | n/a | n/a | 2 | n/a | 2 | 2 | n/a | 2 | 2 | 94 |
| Farina (2020) | 2 | 2 | 2 | 2 | n/a | n/a | n/a | 2 | n/a | 2 | 2 | n/a | 2 | 2 | 100 |
| Farina (2022) | 2 | 2 | 2 | 2 | n/a | n/a | n/a | 2 | 1 | 2 | 1 | n/a | 2 | 2 | 90 |
| Felekoglu (2021) | 2 | 2 | 2 | 2 | n/a | n/a | n/a | 2 | n/a | 2 | 1 | n/a | 2 | 2 | 94 |
| Ferry (2020) | 2 | 2 | 2 | 2 | n/a | n/a | n/a | 2 | n/a | 2 | 1 | n/a | 2 | 1 | 89 |
| Fleming (2016) | 1 | 2 | 2 | 2 | n/a | n/a | n/a | 2 | n/a | 2 | 1 | n/a | 1 | 1 | 78 |
| Froelich (2021) | 2 | 2 | 2 | 2 | n/a | n/a | n/a | 2 | n/a | 2 | 1 | n/a | 2 | 2 | 94 |
| Fuh (2006) | 1 | 1 | 2 | 2 | n/a | n/a | n/a | 2 | n/a | 2 | 1 | n/a | 2 | 2 | 83 |
| Gibbor (2021) | 2 | 2 | 2 | 2 | 2 | 2 | 0 | 2 | 1 | 2 | 2 | 2 | 2 | 2 | 89 |
| Giebel (2014) | 1 | 1 | 2 | 2 | n/a | n/a | n/a | 2 | n/a | 2 | 1 | n/a | 2 | 2 | 83 |
| Giebel (2015) | 2 | 2 | 2 | 2 | n/a | n/a | n/a | 2 | n/a | 2 | 1 | n/a | 2 | 2 | 94 |
| Giebel (2015) | 2 | 1 | 2 | 2 | n/a | n/a | n/a | 2 | n/a | 2 | 2 | n/a | 2 | 2 | 94 |
| Gomez-Gallego (2012) | 1 | 2 | 2 | 1 | n/a | n/a | n/a | 2 | n/a | 2 | 2 | n/a | 2 | 2 | 89 |
| Gomez-Gallego (2015) | 2 | 2 | 2 | 2 | n/a | n/a | n/a | 2 | n/a | 2 | 2 | n/a | 2 | 2 | 100 |
| Gräske (2012) | 2 | 2 | 2 | 2 | n/a | n/a | n/a | 2 | n/a | 2 | 1 | n/a | 2 | 2 | 94 |
| Handels (2018) | 2 | 2 | 2 | 2 | n/a | n/a | n/a | 2 | n/a | 2 | 1 | n/a | 2 | 2 | 94 |
| Heggie (2012) | 1 | 2 | 2 | 2 | n/a | n/a | n/a | 2 | n/a | 2 | 1 | n/a | 2 | 2 | 89 |
| Hessmann (2018) | 2 | 2 | 2 | 2 | n/a | n/a | n/a | 2 | n/a | 2 | 2 | n/a | 2 | 2 | 100 |
| Hilgeman (2014) | 2 | 2 | 2 | 2 | n/a | n/a | n/a | 2 | n/a | 2 | 1 | n/a | 2 | 2 | 94 |
| Huang (2009) | 2 | 2 | 2 | 2 | n/a | n/a | n/a | 2 | n/a | 2 | 1 | n/a | 2 | 2 | 94 |
| Inouye (2010) | 2 | 2 | 2 | 1 | n/a | n/a | n/a | 2 | n/a | 2 | 1 | n/a | 2 | 2 | 89 |
| Jayakody (2023) | 2 | 2 | 1 | 2 | n/a | n/a | n/a | 2 | n/a | 2 | 1 | 1 | 1 | 2 | 80 |
| Jönsson (2006) | 2 | 2 | 2 | 1 | n/a | n/a | n/a | 2 | n/a | 2 | 1 | n/a | 2 | 2 | 89 |
| Kimura (2018) | 2 | 2 | 2 | 2 | n/a | n/a | n/a | 2 | n/a | 2 | 1 | n/a | 2 | 2 | 94 |
| Kimura (2021) | 2 | 2 | 2 | 2 | n/a | n/a | n/a | 2 | n/a | 2 | 2 | n/a | 2 | 2 | 100 |
| Kisvetrova (2018) | 2 | 2 | 2 | 2 | n/a | n/a | n/a | 2 | n/a | 2 | 2 | n/a | 2 | 2 | 100 |
| Kunz (2010) | 1 | 2 | 2 | 2 | n/a | n/a | n/a | 2 | n/a | 2 | 2 | n/a | 2 | 2 | 94 |
| Lacerda (2020) | 2 | 2 | 2 | 2 | n/a | n/a | n/a | 2 | n/a | 2 | 2 | n/a | 2 | 2 | 100 |
| Lacey (2015) | 2 | 2 | 2 | 2 | 2 | 2 | 2 | 2 | 2 | 2 | 2 | 2 | 2 | 2 | 100 |
| Lamb (2018) | 2 | 2 | 2 | 2 | 2 | 2 | n/a | 2 | 2 | 2 | 2 | 2 | 2 | 2 | 100 |
| Lee (2023) | 2 | 1 | 1 | 2 | n/a | n/a | n/a | 2 | 1 | 1 | 2 | n/a | 1 | 2 | 75 |
| Leontjevas (2016) | 2 | 2 | 2 | 2 | n/a | n/a | n/a | 2 | n/a | 2 | 2 | n/a | 2 | 2 | 100 |
| Leroi (2019) | 2 | 2 | 2 | 1 | n/a | n/a | n/a | 2 | n/a | 2 | 1 | n/a | 2 | 2 | 89 |
| Logsdon (1999) | 2 | 2 | 2 | 1 | n/a | n/a | n/a | 2 | n/a | 2 | 1 | n/a | 2 | 2 | 89 |
| Matsui (2006) | 1 | 2 | 2 | 1 | n/a | n/a | n/a | 2 | n/a | 2 | 1 | n/a | 2 | 2 | 83 |
| Moon (2016) | 2 | 2 | 2 | 1 | n/a | n/a | n/a | 2 | n/a | 2 | 2 | n/a | 2 | 2 | 94 |
| Moyle (2012) | 2 | 2 | 2 | 1 | n/a | n/a | n/a | 2 | n/a | 2 | 1 | n/a | 2 | 2 | 89 |
| Naglie (2006) | 2 | 2 | 2 | 2 | n/a | n/a | n/a | 2 | n/a | 2 | 2 | n/a | 2 | 2 | 100 |
| Niikawa (2019) | 2 | 1 | 2 | 2 | n/a | n/a | n/a | 2 | n/a | 2 | 1 | n/a | 2 | 2 | 89 |
| Nogueira (2021) | 2 | 1 | 2 | 2 | n/a | n/a | n/a | 2 | n/a | 2 | 2 | n/a | 2 | 2 | 94 |
| Novelli (2010) | 2 | 1 | 2 | 2 | n/a | n/a | n/a | 2 | n/a | 2 | 1 | n/a | 2 | 2 | 89 |
| O'Shea (2020) | 2 | 2 | 2 | 2 | n/a | n/a | n/a | 2 | n/a | 2 | 2 | n/a | 2 | 2 | 100 |
| Olthof-Nefkens (2023) | 2 | 2 | 1 | 2 | n/a | n/a | n/a | 1 | 1 | 2 | 2 | n/a | 2 | 2 | 85 |
| Orgeta (2015) | 2 | 2 | 2 | 2 | n/a | n/a | n/a | 2 | n/a | 2 | 2 | n/a | 2 | 2 | 100 |
| Phung (2018) | 2 | 2 | 2 | 2 | n/a | n/a | n/a | 2 | n/a | 2 | 2 | n/a | 2 | 2 | 100 |
| Pizzo (2023) | 2 | 2 | 2 | 2 | 1 | 0 | 0 | 2 | 2 | 2 | 2 | 1 | 1 | 2 | 75 |
| Polat (2022) | 2 | 1 | 1 | 1 | n/a | n/a | n/a | 1 | 0 | 1 | 1 | n/a | 1 | 1 | 50 |
| Ready (2004) | 2 | 2 | 2 | 2 | n/a | n/a | n/a | 2 | n/a | 2 | 2 | n/a | 2 | 2 | 100 |
| Rokstad (2017) | 2 | 2 | 2 | 2 | n/a | n/a | n/a | 2 | n/a | 2 | 2 | n/a | 2 | 2 | 100 |
| Rombach (2021) | 2 | 2 | 2 | 2 | n/a | n/a | n/a | 2 | n/a | 2 | 1 | n/a | 2 | 2 | 94 |
| Romhild (2018) | 2 | 2 | 2 | 2 | n/a | n/a | n/a | 2 | n/a | 2 | 2 | n/a | 2 | 2 | 100 |
| Ruggero (2023) | 2 | 1 | 1 | 2 | n/a | n/a | n/a | 2 | 1 | 2 | 1 | n/a | 2 | 2 | 80 |
| Samus (2014) | 2 | 2 | 2 | 2 | 2 | 2 | n/a | 2 | 1 | 2 | 2 | 2 | 2 | 2 | 96 |
| Sands (2004) | 2 | 1 | 2 | 2 | n/a | n/a | n/a | 2 | n/a | 2 | 2 | n/a | 2 | 2 | 94 |
| Santos (2022) | 2 | 2 | 1 | 1 | n/a | n/a | n/a | 1 | n/a | 1 | 1 | n/a | 2 | 2 | 72 |
| Sari (2023) | 2 | 1 | 1 | 2 | n/a | n/a | n/a | 1 | 1 | 2 | 1 | n/a | 2 | 2 | 75 |
| Schuman (2019) | 2 | 1 | 2 | 2 | n/a | n/a | n/a | 2 | n/a | 2 | 2 | n/a | 2 | 2 | 94 |
| Sheehan (2012) | 2 | 1 | 2 | 1 | n/a | n/a | n/a | 2 | n/a | 2 | 1 | n/a | 2 | 2 | 83 |
| Shikimoto (2020) | 1 | 2 | 2 | 1 | n/a | n/a | n/a | 2 | n/a | 1 | 1 | n/a | 1 | 1 | 67 |
| Snow (2005) | 2 | 1 | 2 | 2 | n/a | n/a | n/a | 2 | n/a | 2 | 1 | n/a | 2 | 2 | 89 |
| Sousa (2013) | 2 | 2 | 2 | 1 | n/a | n/a | n/a | 2 | n/a | 2 | 1 | n/a | 2 | 2 | 89 |
| Sousa (2018) | 2 | 2 | 2 | 2 | n/a | n/a | n/a | 2 | n/a | 2 | 1 | n/a | 2 | 2 | 94 |
| Söylemez (2020) | 2 | 2 | 2 | 2 | n/a | n/a | n/a | 2 | n/a | 2 | 2 | n/a | 2 | 2 | 100 |
| Tay (2014) | 2 | 1 | 2 | 2 | n/a | n/a | n/a | 2 | n/a | 2 | 2 | n/a | 2 | 2 | 94 |
| Torrison (2016) | 2 | 2 | 2 | 2 | n/a | n/a | n/a | 2 | n/a | 2 | 1 | n/a | 2 | 2 | 94 |
| Trigg (2015) | 2 | 2 | 2 | 2 | n/a | n/a | n/a | 2 | n/a | 2 | 1 | n/a | 2 | 2 | 94 |
| van Santen (2020) | 2 | 2 | 2 | 2 | 2 | n/a | n/a | 2 | 2 | 2 | 2 | 2 | 2 | 2 | 100 |
| Wolak (2009) | 2 | 2 | 2 | 2 | n/a | n/a | n/a | 2 | n/a | 2 | 1 | n/a | 2 | 2 | 94 |
| Wu (2020) | 2 | 2 | 2 | 2 | n/a | n/a | n/a | 2 | n/a | 2 | 1 | n/a | 2 | 2 | 94 |
| Yamada (2020) | 2 | 2 | 2 | 2 | n/a | n/a | n/a | 2 | n/a | 2 | 1 | n/a | 2 | 2 | 94 |
| Yeaman (2012) | 2 | 1 | 2 | 1 | n/a | n/a | n/a | 2 | n/a | 1 | 1 | n/a | 2 | 2 | 78 |
| Yu (2013) | 2 | 2 | 2 | 1 | n/a | n/a | n/a | 2 | n/a | 2 | 1 | n/a | 2 | 2 | 89 |
| Zhao (2011) | 2 | 2 | 2 | 2 | n/a | n/a | n/a | 2 | n/a | 2 | 2 | n/a | 2 | 2 | 100 |
| Zucchella (2015) | 2 | 2 | 2 | 2 | n/a | n/a | n/a | 2 | n/a | 2 | 1 | n/a | 2 | 2 | 94 |

'2' (yes, item sufficiently addressed), '1' (item partially addressed), '0' (no, not addressed), or 'n/a' (not applicable)

**Q1** Question / objective sufficiently described?

**Q2** Study design evident and appropriate?

**Q3** Method of subject/comparison group selection or source of information/input variables described and appropriate?

**Q4** Subject (and comparison group, if applicable) characteristics sufficiently described?

**Q5** If interventional and random allocation was possible, was it described?

**Q6** If interventional and blinding of investigators was possible, was it reported?

**Q7** If interventional and blinding of subjects was possible, was it reported?

**Q8** Outcome and (if applicable) exposure measure(s) well defined and robust to measurement / misclassification bias? Means of assessment reported?

**Q9** Sample size appropriate?

**Q10** Analytic methods described/justified and appropriate?

**Q11** Some estimate of variance is reported for the main results?

**Q12** Controlled for confounding?

**Q13** Results reported in sufficient detail?

**Q14** Conclusions supported by the results?

**Appendix 5:** **Pooled raw mean difference by proxy perspective and QOL domains**

|  | **Proxy-proxy** | | | |  |  | **Proxy-patient** | | | |  |
| --- | --- | --- | --- | --- | --- | --- | --- | --- | --- | --- | --- |
|  | N* | Self | Proxy | MD | I^2^ |  | N* | Self | Proxy | MD | I^2^ |
| QOL-AD |  |  |  |  |  |  |  |  |  |  |  |
| Physical health | 18 (25) | 2.44 | 2.31 | 0.141 | 57.13 |  | 1 (1) | 2.84 | 2.8 | 0.040 | - |
| Energy | 18 (25) | 2.34 | 2.06 | 0.289 | 76.88 |  | 1 (1) | 2.7 | 2.12 | 0.580 | - |
| Mood | 18 (25) | 2.47 | 2.56 | 0.198 | 73.27 |  | 1 (1) | 2.82 | 2.39 | 0.430 | - |
| Living situation | 18 (25) | 2.88 | 2.76 | 0.085 | 93.22 |  | 1 (1) | 3.31 | 3.19 | 0.120 | - |
| Memory | 18 (25) | 2.08 | 1.78 | 0.342 | 91.05 |  | 1 (1) | 2.26 | 1.43 | 0.830 | - |
| Family | 18 (25) | 2.98 | 2.78 | 0.184 | 80.11 |  | 1 (1) | 3.39 | 3.12 | 0.270 | - |
| Marriage | 18 (25) | 2.85 | 2.68 | 0.152 | 62.28 |  | 1 (1) | 3.42 | 3.16 | 0.260 | - |
| Friends | 18 (25) | 2.68 | 2.42 | 0.250 | 75.48 |  | 1 (1) | 3 | 2.61 | 0.390 | - |
| Self as a whole | 18 (25) | 2.51 | 2.32 | 0.158 | 79.91 |  | 1 (1) | 2.81 | 2.63 | 0.180 | - |
| Chores | 18 (25) | 2.43 | 1.98 | 0.443 | 76.80 |  | 1 (1) | 2.69 | 2.05 | 0.401 | - |
| Things for fun | 18 (25) | 2.33 | 1.99 | 0.334 | 81.11 |  | 1 (1) | 3.06 | 2.68 | 0.380 | - |
| Money | 17 (23) | 2.45 | 2.40 | 0.077 | 79.49 |  | 1 (1) | 2.66 | 2.44 | 0.220 | - |
| Life as a whole | 17 (23) | 2.61 | 2.38 | 0.229 | 79 |  | 1 (1) | 3.06 | 2.73 | 0.330 | - |

**References**

1. Aguirre E., Kang S., Hoare Z., Edwards R.T., and Orrell M. (2016). How does the EQ-5D perform when measuring quality of life in dementia against two other dementia-specific outcome measures? *Qual Life Res*, 25(1):45-9.

2. Aleixo M.A.R., de Borges M.B., Gherman B.R., Teixeira I.A., Neto J.P.S., Santos R.L., Dourado M.C.N., and Marinho V. (2022). Active music therapy in dementia: results from an open-label trial*.* *Jornal Brasileiro de Psiquiatria*, 71(2):117-125.

3. Andrieu S., Coley N., Rolland Y., Cantet C., Arnaud C., Guyonnet S., Nourhashemi F., Grand A., Vellas B., and group P. (2016). Assessing Alzheimer's disease patients' quality of life: Discrepancies between patient and caregiver perspectives*.* *Alzheimers Dement*, 12(4):427-37.

4. Arons A.M., Krabbe P.F., Scholzel-Dorenbos C.J., van der Wilt G.J., and Rikkert M.G. (2013). Quality of life in dementia: a study on proxy bias*.* *BMC Med Res Methodol*, 13:110.

5. Ascher-Svanum H., Chen Y.F., Hake A., Kahle-Wrobleski K., Schuster D., Kendall D., and Heine R.J. (2015). Cognitive and Functional Decline in Patients With Mild Alzheimer Dementia With or Without Comorbid Diabetes*.* *Clin Ther*, 37(6):1195-205.

6. Baptista M.A.T., Santos R.L., Kimura N., Marinho V., Simoes J.P., Laks J., Johannenssen A., Barca M.L., Engedal K., and Dourado M.C.N. (2019). Differences in Awareness of Disease Between Young-onset and Late-onset Dementia*.* *Alzheimer Dis Assoc Disord*, 33(2):129-135.

7. Barbe C., Jolly D., Morrone I., Wolak-Thierry A., Drame M., Novella J.L., and Mahmoudi R. (2018). Factors associated with quality of life in patients with Alzheimer's disease*.* *BMC Geriatr*, 18(1):159.

8. Barrios H., Verdelho A., Narciso S., Goncalves-Pereira M., Logsdon R., and de Mendonca A. (2013). Quality of life in patients with cognitive impairment: validation of the Quality of Life-Alzheimer's Disease scale in Portugal*.* *Int Psychogeriatr*, 25(7):1085-96.

9. Beerens H.C., Sutcliffe C., Renom-Guiteras A., Soto M.E., Suhonen R., Zabalegui A., Bokberg C., Saks K., Hamers J.P., and RightTimePlaceCare C. (2014). Quality of life and quality of care for people with dementia receiving long term institutional care or professional home care: the European RightTimePlaceCare study*.* *J Am Med Dir Assoc*, 15(1):54-61.

10. Belfort T., Simoes J.P., Santos R.L., Lacerda I., and Dourado M.C.N. (2020). Social cognition: Patterns of impairments in mild and moderate Alzheimer's disease*.* *Int J Geriatr Psychiatry*, 35(11):1385-1392.

11. Bosboom P.R., Alfonso H., Eaton J., and Almeida O.P. (2012). Quality of life in Alzheimer's disease: different factors associated with complementary ratings by patients and family carers*.* *Int Psychogeriatr*, 24(5):708-21.

12. Bosboom P.R. and Almeida O.P. (2014). Do changes in specific cognitive functions predict changes in health-related quality of life in people with Alzheimer's disease? *Int J Geriatr Psychiatry*, 29(7):694-703.

13. Bostrom F., Jonsson L., Minthon L., and Londos E. (2007). Patients with dementia with lewy bodies have more impaired quality of life than patients with Alzheimer disease*.* *Alzheimer Dis Assoc Disord*, 21(2):150-4.

14. Brennan S., Doan T., Osada H., and Hashimoto Y. (2023). Validation of the Japanese version of the quality of life-Alzheimer's disease for nursing homes*.* *Aging & Mental Health*, 27(2):281-291.

15. Bruvik F.K., Ulstein I.D., Ranhoff A.H., and Engedal K. (2012). The quality of life of people with dementia and their family carers*.* *Dement Geriatr Cogn Disord*, 34(1):7-14.

16. Crespo M., Hornillos C., and de Quiros M.B. (2013). Factors associated with quality of life in dementia patients in long-term care*.* *Int Psychogeriatr*, 25(4):577-85.

17. Cummings J.L., Raman R., Ernstrom K., Salmon D., Ferris S.H., and Alzheimer's Disease Cooperative Study G. (2006). ADCS Prevention Instrument Project: behavioral measures in primary prevention trials*.* *Alzheimer Dis Assoc Disord*, 20(4 Suppl 3):S147-51.

18. D'Cunha N.M., McKune A.J., Isbel S., Kellett J., Georgousopoulou E.N., and Naumovski N. (2019). Psychophysiological Responses in People Living with Dementia after an Art Gallery Intervention: An Exploratory Study*.* *J Alzheimers Dis*, 72(2):549-562.

19. Dewitte L., Vandenbulcke M., and Dezutter J. (2018). Cognitive functioning and quality of life: Diverging views of older adults with Alzheimer and professional care staff*.* *Int J Geriatr Psychiatry*, 33(8):1074-1081.

20. Dixit D., Spreadbury J., Orlando R., Hayward E., and Kipps C. (2021). Quality of Life Assessments in Individuals With Young-Onset Dementia and Their Caregivers*.* *J Geriatr Psychiatry Neurol*, 34(5):426-433.

21. Dourado M.C.N., Santos R.L., Fischer A., and Mograbi D.C. (2021). Modeling Quality of Life in Alzheimer Disease: The Impact of Cognitive, Functional, and Mood Variables in Self and Carers' Perceptions*.* *J Geriatr Psychiatry Neurol*, 34(6):668-674.

22. Edelman P., Fulton B.R., and Kuhn D. (2004). Comparison of dementia-specific quality of life measures in adult day centers*.* *Home Health Care Serv Q*, 23(1):25-42.

23. Edelman P., Fulton B.R., Kuhn D., and Chang C.H. (2005). A comparison of three methods of measuring dementia-specific quality of life: perspectives of residents, staff, and observers*.* *Gerontologist*, 45 Spec No 1(1):27-36.

24. Farina N., King D., Burgon C., Berwald S., Bustard E., Feeney Y., Habibi R., Comas-Herrera A., Knapp M., Banerjee S., and group M. (2020). Disease severity accounts for minimal variance of quality of life in people with dementia and their carers: analyses of cross-sectional data from the MODEM study*.* *BMC Geriatr*, 20(1):232.

25. Farina N., Hughes L.J., Thomas S., Lowry R.G., and Banerjee S. (2022). The Relationship Between Physical Activity and Health-Related Quality of Life in People With Dementia: An Observational Study*.* *Journal of Aging & Physical Activity*, 30(4):626-634.

26. Felekoglu E., Ozalevli S., Yakut H., Aktan R., and Yener G. (2021). Investigation of the Factors Affecting Quality of Life in Patients with Mild to Moderate Alzheimer's Disease in Terms of Patients and Caregivers*.* *Medicina (Kaunas)*, 57(10).

27. Ferry F., Ryan A., McCauley C.O., Laird E.A., Gibson A., Mulvenna M.D., Bond R., Bunting B., and Curran K. (2020). Economic costs and health-related quality of life associated with individual specific reminiscence: Results from the InspireD Feasibility Study*.* *Dementia (London)*, 19(7):2166-2183.

28. Fleming R., Goodenough B., Low L.F., Chenoweth L., and Brodaty H. (2016). The relationship between the quality of the built environment and the quality of life of people with dementia in residential care*.* *Dementia (London)*, 15(4):663-80.

29. Froelich L., Llado A., Khandker R.K., Pedros M., Black C.M., Sanchez Diaz E.J., Chekani F., and Ambegaonkar B. (2021). Quality of Life and Caregiver Burden of Alzheimer's Disease Among Community Dwelling Patients in Europe: Variation by Disease Severity and Progression*.* *J Alzheimers Dis Rep*, 5(1):791-804.

30. Fuh J.L. and Wang S.J. (2006). Assessing quality of life in Taiwanese patients with Alzheimer's disease*.* *Int J Geriatr Psychiatry*, 21(2):103-7.

31. Gibbor L., Forde L., Yates L., Orfanos S., Komodromos C., Page H., Harvey K., and Spector A. (2021). A feasibility randomised control trial of individual cognitive stimulation therapy for dementia: impact on cognition, quality of life and positive psychology*.* *Aging Ment Health*, 25(6):999-1007.

32. Giebel C.M., Sutcliffe C., Stolt M., Karlsson S., Renom-Guiteras A., Soto M., Verbeek H., Zabalegui A., and Challis D. (2014). Deterioration of basic activities of daily living and their impact on quality of life across different cognitive stages of dementia: a European study*.* *Int Psychogeriatr*, 26(8):1283-93.

33. Giebel C.M., Sutcliffe C., and Challis D. (2015). Activities of daily living and quality of life across different stages of dementia: a UK study*.* *Aging Ment Health*, 19(1):63-71.

34. Giebel C.M., Sutcliffe C., Renom-Guiteras A., Arve S., Hallberg I.R., Soto M., Zabalegui A., Hamers J., Saks K., and Challis D. (2015). Depressive symptomatology in severe dementia in a European sample: prevalence, associated factors and prescription rate of antidepressants*.* *Int Psychogeriatr*, 27(4):657-67.

35. Gomez-Gallego M., Gomez-Amor J., and Gomez-Garcia J. (2012). Determinants of quality of life in Alzheimer's disease: perspective of patients, informal caregivers, and professional caregivers*.* *Int Psychogeriatr*, 24(11):1805-15.

36. Gomez-Gallego M., Gomez-Garcia J., and Ato-Lozano E. (2015). Addressing the bias problem in the assessment of the quality of life of patients with dementia: determinants of the accuracy and precision of the proxy ratings*.* *J Nutr Health Aging*, 19(3):365-72.

37. Graske J., Fischer T., Kuhlmey A., and Wolf-Ostermann K. (2012). Quality of life in dementia care--differences in quality of life measurements performed by residents with dementia and by nursing staff*.* *Aging Ment Health*, 16(7):819-27.

38. Handels R.L.H., Skoldunger A., Bieber A., Edwards R.T., Goncalves-Pereira M., Hopper L., Irving K., Jelley H., Kerpershoek L., Marques M.J., Meyer G., Michelet M., Portolani E., Rosvik J., Selbaek G., Stephan A., de Vugt M., Wolfs C., Woods B., Zanetti O., Verhey F., Wimo A., and Actifcare c. (2018). Quality of Life, Care Resource Use, and Costs of Dementia in 8 European Countries in a Cross-Sectional Cohort of the Actifcare Study*.* *J Alzheimers Dis*, 66(3):1027-1040.

39. Heggie M., Morgan D., Crossley M., Kirk A., Wong P., Karunanayake C., and Beever R. (2012). Quality of life in early dementia: Comparison of rural patient and caregiver ratings at baseline and one year*.* *Dementia (London)*, 11(4):521-541.

40. Hessmann P., Dodel R., Baum E., Muller M.J., Paschke G., Kis B., Zeidler J., Klora M., Reese J.P., and Balzer-Geldsetzer M. (2018). Antidepressant medication in a German cohort of patients with Alzheimer's disease *Int J Clin Pharmacol Ther*, 56(3):101-112.

41. Hilgeman M.M., Allen R.S., Snow A.L., Durkin D.W., DeCoster J., and Burgio L.D. (2014). Preserving Identity and Planning for Advance Care (PIPAC): preliminary outcomes from a patient-centered intervention for individuals with mild dementia*.* *Aging Ment Health*, 18(4):411-24.

42. Huang H.L., Chang M.Y., Tang J.S., Chiu Y.C., and Weng L.C. (2009). Determinants of the discrepancy in patient- and caregiver-rated quality of life for persons with dementia*.* *J Clin Nurs*, 18(22):3107-18.

43. Inouye K., Pedrazzani E.S., Pavarini S.C., and Toyoda C.Y. (2010). Quality of life of elderly with Alzheimer's disease: a comparative study between the patients and the caregivers report*.* *Rev Lat Am Enfermagem*, 18(1):26-32.

44. Jayakody S. and Arambepola C. (2023). Patient and caregiver perspectives on quality of life in dementia: Evidence from a South Asian population*.* *PloS one*, 18(5):e0285701.

45. Jönsson L., Andreasen N., Kilander L., Soininen H., Waldemar G., Nygaard H., Winblad B., Jonhagen M.E., Hallikainen M., and Wimo A. (2006). Patient- and proxy-reported utility in Alzheimer disease using the EuroQoL*.* *Alzheimer Dis Assoc Disord*, 20(1):49-55.

46. Kimura N.R.S., Baptista M.A.T., Santos R.L., Portugal M.D.G., Johannenssen A., Barca M.L., Engedal K., Laks J., Simoes J.P., Rodrigues V.M., and Dourado M.C.N. (2018). Caregivers' Perspectives of Quality of Life of People With Young- and Late-Onset Alzheimer Disease*.* *J Geriatr Psychiatry Neurol*, 31(2):76-83.

47. Kimura N.R.S., Simoes J.P., Santos R.L., Baptista M.A.T., Portugal M.D.G., Johannessen A., Barca M.L., Engedal K., Laks J., Rodrigues V.M., and Dourado M.C.N. (2021). Young- and Late-Onset Dementia: A Comparative Study of Quality of Life, Burden, and Depressive Symptoms in Caregivers*.* *J Geriatr Psychiatry Neurol*, 34(5):434-444.

48. Kisvetrova H., Skoloudik D., Herzig R., Valis M., Juraskova B., Krulova P., Langova K., Bermellova J., and Yamada Y. (2018). Psychometric Validation of the Czech Version of the Quality of Life - Alzheimer's Disease Scale in Patients with Early-Stage Dementia*.* *Dement Geriatr Cogn Disord*, 46(1-2):109-118.

49. Kunz S. (2010). Psychometric properties of the EQ-5D in a study of people with mild to moderate dementia*.* *Qual Life Res*, 19(3):425-34.

50. Lacerda I.B., Santos R.L., Belfort T., Neto J.P.S., and Dourado M.C.N. (2020). Patterns of discrepancies in different objects of awareness in mild and moderate Alzheimer's disease*.* *Aging Ment Health*, 24(5):789-796.

51. Lacey L., Bobula J., Rudell K., Alvir J., and Leibman C. (2015). Quality of Life and Utility Measurement in a Large Clinical Trial Sample of Patients with Mild to Moderate Alzheimer's Disease: Determinants and Level of Changes Observed*.* *Value Health*, 18(5):638-45.

52. Lamb S.E., Mistry D., Alleyne S., Atherton N., Brown D., Copsey B., Dosanjh S., Finnegan S., Fordham B., Griffiths F., Hennings S., Khan I., Khan K., Lall R., Lyle S., Nichols V., Petrou S., Zeh P., and Sheehan B. (2018). Aerobic and strength training exercise programme for cognitive impairment in people with mild to moderate dementia: the DAPA RCT*.* *Health Technol Assess*, 22(28):1-202.

53. Lee D.-C.A., Haines T.P., Callisaya M.L., and Hill K.D. (2023). A Scalable Program for Improving Physical Activity in Older People with Dementia Including Culturally and Linguistically Diverse (CALD) Groups Who Receive Home Support: A Feasibility Study*.* *International Journal of Environmental Research and Public Health*, 20(4).

54. Leontjevas R., Teerenstra S., Smalbrugge M., Koopmans R.T., and Gerritsen D.L. (2016). Quality of life assessments in nursing homes revealed a tendency of proxies to moderate patients' self-reports*.* *J Clin Epidemiol*, 80:123-133.

55. Leroi I., Simkin Z., Hooper E., Wolski L., Abrams H., Armitage C.J., Camacho E., Charalambous A.P., Collin F., Constantinidou F., Dawes P., Elliott R., Falkingham S., Frison E., Hann M., Helmer C., Himmelsbach I., Hussain H., Marie S., Montecelo S., Thodi C., and Yeung W.K. (2020). Impact of an intervention to support hearing and vision in dementia: The SENSE-Cog Field Trial*.* *Int J Geriatr Psychiatry*, 35(4):348-357.

56. Logsdon R.G., Gibbons L.E., McCurry S.M., and Teri L. (1999). Quality of life in Alzheimer's disease: Patient and caregiver reports*.* *Journal of Mental Health and Aging*, 5:21-32.

57. Matsui T., Nakaaki S., Murata Y., Sato J., Shinagawa Y., Tatsumi H., and Furukawa T.A. (2006). Determinants of the quality of life in Alzheimer's disease patients as assessed by the Japanese version of the Quality of Life-Alzheimer's disease scale*.* *Dement Geriatr Cogn Disord*, 21(3):182-91.

58. Moon H., Townsend A.L., Dilworth-Anderson P., and Whitlatch C.J. (2016). Predictors of Discrepancy Between Care Recipients With Mild-to-Moderate Dementia and Their Caregivers on Perceptions of the Care Recipients' Quality of Life*.* *Am J Alzheimers Dis Other Demen*, 31(6):508-15.

59. Moyle W., Murfield J.E., Griffiths S.G., and Venturato L. (2012). Assessing quality of life of older people with dementia: a comparison of quantitative self-report and proxy accounts*.* *J Adv Nurs*, 68(10):2237-46.

60. Naglie G., Tomlinson G., Tansey C., Irvine J., Ritvo P., Black S.E., Freedman M., Silberfeld M., and Krahn M. (2006). Utility-based Quality of Life measures in Alzheimer's disease*.* *Qual Life Res*, 15(4):631-43.

61. Niikawa H., Kawano Y., Yamanaka K., Okamura T., Inagaki H., Ito K., and Awata S. (2019). Reliability and validity of the Japanese version of a self-report (DEMQOL) and carer proxy (DEMQOL-PROXY) measure of health-related quality of life in people with dementia*.* *Geriatr Gerontol Int*, 19(6):487-491.

62. Nogueira M.M.L., Neto J.P.S., and Dourado M.C.N. (2021). Quality of Life of People With Alzheimer Disease: Comparison Between Dyads Degree of Kinship*.* *J Geriatr Psychiatry Neurol*, 34(2):119-127.

63. Novelli M.M., Nitrini R., and Caramelli P. (2010). Validation of the Brazilian version of the quality of life scale for patients with Alzheimer's disease and their caregivers (QOL-AD)*.* *Aging Ment Health*, 14(5):624-31.

64. O'Shea E., Hopper L., Marques M., Goncalves-Pereira M., Woods B., Jelley H., Verhey F., Kerpershoek L., Wolfs C., de Vugt M., Stephan A., Bieber A., Meyer G., Wimo A., Michelet M., Selbaek G., Portolani E., Zanetti O., Irving K., and Actifcare C. (2020). A comparison of self and proxy quality of life ratings for people with dementia and their carers: a European prospective cohort study*.* *Aging Ment Health*, 24(1):162-170.

65. Olthof-Nefkens M.W.L.J., Derksen E.W.C., Lambregts B., de Swart B.J.M., Nijhuis-van der Sanden M.W.G., and Kalf J.G. (2023). Clinimetric Evaluation of the Experienced Communication in Dementia Questionnaire*.* *The Gerontologist*, 63(1):40-51.

66. Orgeta V., Edwards R.T., Hounsome B., Orrell M., and Woods B. (2015). The use of the EQ-5D as a measure of health-related quality of life in people with dementia and their carers*.* *Qual Life Res*, 24(2):315-24.

67. Phung T.K.T., Siersma V., Vogel A., Waldorff F.B., and Waldemar G. (2018). Self-rated versus Caregiver-rated Health for Patients with Mild Dementia as Predictors of Patient Mortality*.* *Am J Geriatr Psychiatry*, 26(3):375-385.

68. Pizzo E., Wenborn J., Burgess J., Mundy J., Orrell M., King M., Omar R., and Morris S. (2022). Cost-utility analysis of community occupational therapy in dementia (COTiD-UK) versus usual care: Results from VALID, a multi-site randomised controlled trial in the UK*.* *PloS one*, 17(2):e0262828.

69. Polat B.S.A. and Karadas O. (2022). Evaluation of a computer-based cognitive training program for early-stage Alzheimer's disease*.* *Annals of Clinical and Analytical Medicine*, 13(2):175-179.

70. Ready R.E., Ott B.R., and Grace J. (2004). Patient versus informant perspectives of Quality of Life in Mild Cognitive Impairment and Alzheimer's disease*.* *Int J Geriatr Psychiatry*, 19(3):256-65.

71. Rokstad A.M., Engedal K., Kirkevold O., Saltyte Benth J., Barca M.L., and Selbaek G. (2017). The association between attending specialized day care centers and the quality of life of people with dementia*.* *Int Psychogeriatr*, 29(4):627-636.

72. Rombach I., Iftikhar M., Jhuti G.S., Gustavsson A., Lecomte P., Belger M., Handels R., Castro Sanchez A.Y., Kors J., Hopper L., Olde Rikkert M., Selbaek G., Stephan A., Sikkes S.A.M., Woods B., Goncalves-Pereira M., Zanetti O., Ramakers I., Verhey F.R.J., Gallacher J., Actifcare C., Le A.R.N.C., Landeiro F., Gray A.M., and Consortium R. (2021). Obtaining EQ-5D-5L utilities from the disease specific quality of life Alzheimer's disease scale: development and results from a mapping study*.* *Qual Life Res*, 30(3):867-879.

73. Romhild J., Fleischer S., Meyer G., Stephan A., Zwakhalen S., Leino-Kilpi H., Zabalegui A., Saks K., Soto-Martin M., Sutcliffe C., Rahm Hallberg I., Berg A., and RightTimePlaceCare C. (2018). Inter-rater agreement of the Quality of Life-Alzheimer's Disease (QoL-AD) self-rating and proxy rating scale: secondary analysis of RightTimePlaceCare data*.* *Health Qual Life Outcomes*, 16(1):131.

74. Ruggero L., Croot K., and Nickels L. (2023). Quality of Life Ratings and Proxy Bias in Primary Progressive Aphasia: Two Sides to the Story? *American journal of Alzheimer's disease and other dementias*, 38:15333175231177668.

75. Samus Q.M., Johnston D., Black B.S., Hess E., Lyman C., Vavilikolanu A., Pollutra J., Leoutsakos J.M., Gitlin L.N., Rabins P.V., and Lyketsos C.G. (2014). A multidimensional home-based care coordination intervention for elders with memory disorders: the maximizing independence at home (MIND) pilot randomized trial*.* *Am J Geriatr Psychiatry*, 22(4):398-414.

76. Sands L.P., Ferreira P., Stewart A.L., Brod M., and Yaffe K. (2004). What explains differences between dementia patients' and their caregivers' ratings of patients' quality of life? *Am J Geriatr Psychiatry*, 12(3):272-80.

77. Santos R.L., Simões Neto J.P., Belfort T., Lacerda I.B., and Dourado M.C.N. (2022). Patterns of impairment in decision-making capacity in Alzheimer's disease and its relationship with cognitive and clinical variables*.* *Revista brasileira de psiquiatria (Sao Paulo, Brazil : 1999)*, 44(3):271-278.

78. Sari Y.M., Burton E., Lee D.-C.A., and Hill K.D. (2023). A Telehealth Home-Based Exercise Program for Community-Dwelling Older People with Dementia in Indonesia: A Feasibility Study*.* *International Journal of Environmental Research and Public Health*, 20(4).

79. Schumann C., Alexopoulos P., and Perneczky R. (2019). Determinants of self- and carer-rated quality of life and caregiver burden in Alzheimer disease*.* *Int J Geriatr Psychiatry*, 34(10):1378-1385.

80. Sheehan B.D., Lall R., Stinton C., Mitchell K., Gage H., Holland C., and Katz J. (2012). Patient and proxy measurement of quality of life among general hospital in-patients with dementia*.* *Aging Ment Health*, 16(5):603-7.

81. Shikimoto R., Nakaaki S., Sato J., Sato H., and Mimura M. (2020). Comparison of various items of the quality of life in Alzheimer's disease scale (QOL-AD) in patients with mild or moderate stages of dementia: a cross-sectional study*.* *Psychogeriatrics*, 20(6):926-928.

82. Snow A.L., Dani R., Souchek J., Sullivan G., Ashton C.M., and Kunik M.E. (2005). Comorbid psychosocial symptoms and quality of life in patients with dementia*.* *Am J Geriatr Psychiatry*, 13(5):393-401.

83. Sousa M.F., Santos R.L., Arcoverde C., Simoes P., Belfort T., Adler I., Leal C., and Dourado M.C. (2013). Quality of life in dementia: the role of non-cognitive factors in the ratings of people with dementia and family caregivers*.* *Int Psychogeriatr*, 25(7):1097-105.

84. Sousa M.F.B., Santos R.L., Simoes P., Conde-Sala J.L., and Dourado M.C.N. (2018). Discrepancies Between Alzheimer's Disease Patients' and Caregivers' Ratings About Patients' Quality of Life: A 1-year Observation Study in Brazil*.* *Alzheimer Dis Assoc Disord*, 32(3):240-246.

85. Söylemez B.A., Kucukguclu O., Akyol M.A., and Isik A.T. (2020). Quality of life and factors affecting it in patients with Alzheimer's disease: a cross-sectional study*.* *Health Qual Life Outcomes*, 18(1):304.

86. Tay L., Chua K.C., Chan M., Lim W.S., Ang Y.Y., Koh E., and Chong M.S. (2014). Differential perceptions of quality of life (QoL) in community-dwelling persons with mild-to-moderate dementia*.* *Int Psychogeriatr*, 26(8):1273-82.

87. Torisson G., Stavenow L., Minthon L., and Londos E. (2016). Reliability, validity and clinical correlates of the Quality of Life in Alzheimer's disease (QoL-AD) scale in medical inpatients*.* *Health Qual Life Outcomes*, 14:90.

88. Trigg R., Jones R.W., Knapp M., King D., Lacey L.A., and Groups D.-I. (2015). The relationship between changes in quality of life outcomes and progression of Alzheimer's disease: results from the dependence in AD in England 2 longitudinal study*.* *Int J Geriatr Psychiatry*, 30(4):400-8.

89. van Santen J., Droes R.M., Twisk J.W.R., Blanson Henkemans O.A., van Straten A., and Meiland F.J.M. (2020). Effects of Exergaming on Cognitive and Social Functioning of People with Dementia: A Randomized Controlled Trial*.* *J Am Med Dir Assoc*, 21(12):1958-1967 e5.

90. Wolak A., Novella J.L., Drame M., Guillemin F., Di Pollina L., Ankri J., Aquino J.P., Morrone I., Blanchard F., and Jolly D. (2009). Transcultural adaptation and psychometric validation of a French-language version of the QoL-AD*.* *Aging Ment Health*, 13(4):593-600.

91. Wu Y.T., Nelis S.M., Quinn C., Martyr A., Jones I.R., Victor C.R., Knapp M., Henderson C., Hindle J.V., Jones R.W., Kopelman M.D., Morris R.G., Pickett J.A., Rusted J.M., Thom J.M., Litherland R., Matthews F.E., Clare L., and team I.P. (2020). Factors associated with self- and informant ratings of quality of life, well-being and life satisfaction in people with mild-to-moderate dementia: results from the Improving the experience of Dementia and Enhancing Active Life programme*.* *Age Ageing*, 49(3):446-452.

92. Yamada T., Nakaaki S., Sato J., Sato H., Shikimoto R., Furukawa T.A., Mimura M., and Akechi T. (2020). Factor structure of the Japanese version of the Quality of Life in Alzheimer's Disease Scale (QOL-AD)*.* *Psychogeriatrics*, 20(1):79-86.

93. Yeaman P.A., Kim D.Y., Alexander J.L., Ewing H., and Kim K.Y. (2013). Relationship of physical and functional independence and perceived quality of life of veteran patients with Alzheimer disease*.* *Am J Hosp Palliat Care*, 30(5):462-6.

94. Yu H.M., He R.L., Ai Y.M., Liang R.F., and Zhou L.Y. (2013). Reliability and validity of the quality of life-Alzheimer disease Chinese version*.* *J Geriatr Psychiatry Neurol*, 26(4):230-6.

95. Zhao H., Novella J.L., Drame M., Mahmoudi R., Barbe C., di Pollina L., Aquino J.P., Pfitzenmeyer P., Rouaud O., George M.Y., Ankri J., Blanchard F., and Jolly D. (2012). Factors associated with caregivers' underestimation of quality of life in patients with Alzheimer's disease*.* *Dement Geriatr Cogn Disord*, 33(1):11-7.

96. Zucchella C., Bartolo M., Bernini S., Picascia M., and Sinforiani E. (2015). Quality of life in Alzheimer disease: a comparison of patients' and caregivers' points of view*.* *Alzheimer Dis Assoc Disord*, 29(1):50-4.
